# Phase I/II Study of AXL-Specific Antibody–Drug Conjugate Enapotamab Vedotin in Patients with Advanced Solid Tumors

**DOI:** 10.1158/2767-9764.CRC-25-0359

**Published:** 2025-11-26

**Authors:** Kristoffer Staal Rohrberg, Juanita S. Lopez, Mohammed M. Milhem, Christian U. Blank, Irene Reijers, Fiona Thistlethwaite, Ruth Plummer, Sarina A. Piha-Paul, Pasi A. Jänne, Elaine Shum, Heather M. Shaw, Philip R. Debruyne, Cristopher Lao, Jean-Francois Baurain, Jennifer H. Choe, Eelke Gort, Yujie Zhao, Guy Jerusalem, Patrick Schöffski, Andrew William Chen, Eric A. Cohen, Walter C. Mankowski, Leonid Roshkovan, Sharyn I. Katz, Despina Kontos, Lauren K. Brady, Mohammed Qutaish, Patricia Garrido Castro, Nora Pencheva, Gaurav Bajaj, Yali Fu, Kristian Windfeld, Panagiota Reiter, Maria Jure-Kunkel, Brandon W. Higgs, Katayoun I. Amiri, Tahamtan Ahmadi, Ulf Forssmann, Suresh S. Ramalingam, Ignace Vergote

**Affiliations:** 1 Department of Oncology, Copenhagen University Hospital (Rigshospitalet), Copenhagen, Denmark.; 2 Drug Development Unit, Royal Marsden Hospital/Institute of Cancer Research, Sutton, United Kingdom.; 3 Department of Internal Medicine, Carver College of Medicine, University of Iowa, Iowa City, Iowa.; 4 Division of Molecular Oncology and Immunology, Department of Medical Oncology, Netherlands Cancer Institute (NKI), Amsterdam, the Netherlands.; 5 Department of Medical Oncology, Leiden University Medical Center (LUMC), Leiden, the Netherlands.; 6 Department of Hematology and Oncology, University Clinic Regensburg (UKR), Regensburg, Germany.; 7 The Christie NHS Foundation Trust and Division of Cancer Sciences, The University of Manchester, Manchester, United Kingdom.; 8 Newcastle University, Newcastle upon Tyne, United Kingdom.; 9 Department of Investigational Cancer Therapeutics, The University of Texas MD Anderson Cancer Center, Houston, Texas.; 10 Lowe Center for Thoracic Oncology, Dana-Farber Cancer Institute, Boston, Massachusetts.; 11 Medical Oncology, Laura and Isaac Perlmutter Cancer Center, NYU Langone Health, New York, New York.; 12 National Institute for Health Research (NIHR) University College London Hospitals (UCLH) Clinical Research Facility, National Health Service (NHS) Foundation Trust, London, United Kingdom.; 13 Department of Medical Oncology, Mount Vernon Cancer Centre, Northwood, United Kingdom.; 14 Department of Medical Oncology, Kortrijk Cancer Centre, AZ Groeninge, Kortrijk, Belgium.; 15 Medical Technology Research Centre (MTRC), School of Allied Health and Social Care, Faculty of Health, Medicine and Social Care, Anglia Ruskin University, Chelmsford, United Kingdom.; 16 School of Nursing and Midwifery, Faculty of Health, University of Plymouth, Plymouth, United Kingdom.; 17 Division of Hematology Oncology, Department of Internal Medicine, Rogel Cancer Center, Ann Arbor, Michigan.; 18 Department of Medical Oncology, Institut Roi Albert II, Cliniques Universitaires Saint-Luc, UC Louvain and Institute for Experimental and Clinical Research, Université Catholique de Louvain, Brussels, Belgium.; 19 Division of Hematology and Oncology, Department of Medicine, Vanderbilt-Ingram Cancer Center, Vanderbilt University Medical Center, Nashville, Tennessee.; 20 Department of Medical Oncology, UMC Utrecht Cancer Center, Utrecht, the Netherlands.; 21 Department of Hematology and Oncology, Mayo Clinic, Jacksonville, Florida.; 22 Medical Oncology Department, CHU Liège and Liège University, Liege, Belgium.; 23 General Medical Oncology, University Hospitals Leuven, Leuven, Belgium.; 24 Department of Radiology, Perelman School of Medicine, University of Pennsylvania, Philadelphia, Pennsylvania.; 25 Department of Radiology, Columbia University, New York, New York.; 26 Genmab US., Plainsboro, New Jersey.; 27 Genmab B.V., Utrecht, the Netherlands.; 28 Genmab A/S, Copenhagen, Denmark.; 29 Department of Hematology and Medical Oncology, Winship Cancer Institute of Emory, Emory University School of Medicine, Atlanta, Georgia.; 30 Department of Gynecology and Obstetrics, Gynecologic Oncology, Leuven Cancer Institute, Catholic University Leuven, Leuven, Belgium.

## Abstract

**Purpose:**

AXL, a receptor tyrosine kinase related to oncogenic processes, is aberrantly expressed in various cancers and associated with treatment resistance. Enapotamab vedotin (EnaV), a novel anti-AXL human IgG1 and monomethyl auristatin E antibody–drug conjugate, demonstrated antitumor activity in preclinical models, including non–small cell lung cancer (NSCLC). This phase 1/2 study assessed the safety and preliminary efficacy of EnaV in solid tumors.

**Patients and Methods:**

This study comprised dose-escalation and dose-expansion phases; both phases investigated EnaV once every 3 weeks (Q3W) and EnaV on days 1, 8, and 15 of a 28-day cycle (3Q4W). Primary objectives determined the maximum tolerated dose (dose escalation) and safety (dose expansion). Pharmacokinetic profile, antitumor activity, and AXL expression were also assessed.

**Results:**

During dose escalation, 32 patients received EnaV Q3W; 15 received EnaV 3Q4W. The maximum tolerated dose and recommended phase 2 dose were 2.2 mg/kg in Q3W and 1.0 mg/kg in 3Q4W schedules. In dose expansion, 189 patients received EnaV Q3W; 70 received EnaV 3Q4W. Common adverse events in dose expansion included fatigue, constipation, nausea, decreased appetite, and diarrhea. Overall response rates ranged from 4.5% to 12.5% with Q3W dose schedule and from 9.1% to 11.5% with 3Q4W dose schedule. Disease control rates for NSCLC cohorts were 40.9% to 50.0%. NSCLC subset analysis demonstrated correlation between radiomics signature and disease control. The relationship between clinical activity and AXL expression was not apparent.

**Conclusions:**

EnaV had an acceptable safety profile; however, because the evaluation of antitumor activity did not show clinically meaningful responses, clinical development of EnaV was discontinued.

**Significance:**

EnaV, an anti-AXL human IgG1 and monomethyl auristatin E antibody–drug conjugate, showed single-agent antitumor activity in preclinical models. This phase 1/2 study of EnaV demonstrated a manageable safety profile and antitumor activity in selected tumor types. Further studies exploring alternative targeting modalities, patient selection, and/or combinations are needed.

## Introduction

AXL is a member of the TYRO3, AXL, and MERTK (TAM)-family receptor tyrosine kinase (RTK) located upstream of various biological processes, including cell proliferation, differentiation, and migration; the *AXL* gene has been shown to be oncogenic in a range of cancers, including non–small cell lung cancer (NSCLC) and melanoma (1, 2). Overexpression, or increased activation of AXL, is associated with epithelial-to-mesenchymal transition in preclinical models and tumor metastasis and poor clinical prognosis in patients with various cancer types (1–3). In addition, upregulated AXL expression has been associated with acquired resistance to various anticancer agents, including tyrosine kinase inhibitor (TKI) and checkpoint inhibitor therapy (1, 3–8). Cross-talk between AXL and other RTKs, such as EGFR, may reduce the effectiveness of TKIs (4, 5, 9). Furthermore, evidence suggests that AXL expression in tumor cells is associated with an immunosuppressive phenotype that may be associated with resistance to PD-1 blockade (6, 7). AXL therefore represents a potential therapeutic target for treatment of patients with solid tumors, including those with disease progression following treatment with TKIs and immunotherapy.

Antibody–drug conjugates (ADC) have become an important tool for treatment of cancer, with at least 14 ADCs receiving market approval worldwide (10). ADCs comprise an antibody targeting a tumor-specific antigen linked to a chemotherapeutic payload, which allows for a more targeted, and potentially better tolerated, approach to chemotherapy (10). Monomethyl auristatin E (MMAE)—referred to as vedotin in ADC nomenclature—is a microtubule-disrupting agent that has been successfully used as a component of a number of approved ADCs, such as brentuximab vedotin, tisotumab vedotin, enfortumab vedotin, and polatuzumab vedotin (11–14). Enapotamab vedotin (EnaV) is an experimental ADC composed of an anti-AXL human monoclonal IgG1-kappa antibody (HuMax-AXL) conjugated via a valine–citrulline linker to MMAE (15, 16). Upon binding to AXL, EnaV is rapidly internalized and trafficked to lysosomes, which proteolytically cleave the valine–citrulline linker and release MMAE from the ADC complex (17). Although the mechanism of action of EnaV is dependent on AXL expression, it acts independently of activation of AXL/Gas6 signaling (15), with tumor cell death being driven by MMAE interference with cell division. EnaV has shown promising antitumor activity in preclinical evaluation, including in models of soft-tissue sarcoma and NSCLC, as well as in immunotherapy-resistant models of melanoma and NSCLC (16–18). Here, we share key results from the first-in-human trial evaluating the safety and preliminary efficacy of EnaV in multiple cohorts of patients with solid tumors in which the use of systemic tubulin inhibitors is standard of care. This trial leveraged two dosing regimens to evaluate possible differences in safety and efficacy that might arise as a result of different pharmacokinetic (PK) profiles. The specific populations studied were selected based on expected intrinsic high tumor expression of AXL (e.g., sarcoma) or hypothesized increased AXL tumor expression upon development of drug resistance in prior lines of therapy (e.g., relapsed/refractory NSCLC and melanoma).

## Patients and Methods

### Study design and treatment

This open-label, multicenter, phase 1/2, first-in-human trial (clinicaltrials.gov: NCT02988817) consisted of a dose-escalation phase and a dose-expansion phase. The dose-escalation phase included adults with relapsed or refractory cancer of the ovary, cervix, endometrium, thyroid, NSCLC, or melanoma (cutaneous mucosal, acral, or uveal melanoma) who were not candidates for further standard therapy. Two treatment schedules of EnaV administration were assessed: a 21-day schedule, with a single dose administered intravenously over a 3-week period (Q3W), and a 28-day schedule with three weekly doses followed by a 1-week drug holiday over a 4-week period (3Q4W) (Supplementary Fig. S1). The Q3W dose schedule was initiated based on single patient cohorts for the initial EnaV doses of 0.3 mg/kg and 0.6 mg/kg. Subsequent dose escalation used a Bayesian continual reassessment model for the Q3W group with planned intervals up to a maximum of 2.8 mg/kg Q3W until the maximum tolerated dose (MTD) and/or recommended phase 2 dose (RP2D) was identified. When a minimum of eight patients had been treated and evaluated for dose-limiting toxicities (DLT), and the 1.5 mg/kg dose level declared safe for the Q3W dose schedule, dose escalation in the 3Q4W dose schedule group was initiated and used a 3 + 3 design with an optimized range of planned doses (0.6–1.2 mg/kg).

The dose-expansion phase was designed to provide further data on the safety, tolerability, and efficacy of the selected RP2D. The populations enrolled in the dose-expansion cohorts are shown in Table 1. Briefly, cohorts 1, 2, and 8 included previously treated patients with advanced or metastatic NSCLC who received EnaV 1.8 mg/kg or 2.2 mg/kg Q3W or 1.0 mg/kg 3Q4W. Cohorts 3 and 4 included previously treated patients with advanced or metastatic melanoma who received EnaV 2.2 mg/kg Q3W. Cohort 5 included previously treated patients with advanced or metastatic sarcoma who received EnaV 2.2 mg/kg Q3W. Cohort 6 included patients with metastatic solid tumors (excluding NSCLC, melanoma, sarcoma, and ovarian cancer unless a known *AXL* gene amplification was present) whose disease progressed following PD-1/PD-L1 inhibitor therapy for metastatic disease as the last prior treatment and received EnaV 1.0 mg/kg 3Q4W. Cohort 7 included patients with platinum-resistant ovarian cancer who received EnaV 2.2 mg/kg Q3W.

**Table 1.** Study cohorts in the dose-expansion phase.

Cohort	Tumor type	Biomarkers	Other entry criteria	EnaV dosing schedule
1	NSCLC	Sensitizing EGFR mutations and/or mutations targeted by third-generation TKIs	≤4 prior systemic treatments; EGFR inhibitor, PD-1/PD-L1 inhibitor, or platinum-doublet as the most recent therapy	2.2 mg/kg Q3W
2	NSCLC	No activating *EGFR* mutations or *ALK* rearrangements	≤2 prior lines of therapy, which included an *EGFR* inhibitor, PD-1/PD-L1 inhibitor, or platinum-based chemotherapy	1.8 mg/kg or 2.2 mg/kg Q3W
3	Melanoma	*BRAF* V600 mutation	≤4 prior systemic treatments; *BRAF* inhibitor or checkpoint inhibitor as the most recent therapy	2.2 mg/kg Q3W
4	Melanoma	*BRAF* V600 wild-type	Up to three prior systemic treatments; the last treatment should have been a checkpoint inhibitor	2.2 mg/kg Q3W
5	Sarcoma	—	≤3 prior systemic treatments. Limited to undifferentiated pleomorphic sarcoma, liposarcoma, leiomyosarcoma, synovial sarcoma, Ewing sarcoma, osteosarcoma, and chondrosarcoma	2.2 mg/kg Q3W
6	Metastatic solid tumor^a^	—	Failed prior treatment with a PD-1/PD-L1 inhibitor; immune checkpoint inhibitor as the most recent therapy	1.0 mg/kg 3Q4W
7	Ovarian cancer	—	Platinum-resistant; failed ≥2 prior systemic therapies but not more than five for recurrent disease	2.2 mg/kg Q3W
8	NSCLC	No activating *EGFR* mutations or *ALK* rearrangements	≤2 prior lines of therapy, including an *EGFR* inhibitor, PD-1/PD-L1 inhibitor, or platinum-based chemotherapy	1.0 mg/kg 3Q4W

a Excluding NSCLC, melanoma, sarcoma, and ovarian cancer unless a known *AXL* gene amplification was present.

Prophylactic constipation-mitigation measures included administration of oral bisacodyl (starting dose, 5 mg once daily) or oral docusate sodium (if bisacodyl was unavailable; starting dose, 100 mg twice daily) on day 5 following administration of EnaV until day 21 for the Q3W dose schedule and until day 28 for the 3Q4W dose schedule, with dose adjustments as required. Patients were given dietary guidance to prevent constipation and a patient diary for recording stool frequency, consistency, and use of prophylactic treatment. The patient diary also contained guidance on modification of the prophylactic treatment dosing as needed.

The study was approved by an Independent Ethics Committee or Institutional Review Board at each participating site. The trial was conducted in accordance with the International Council for Harmonization E6 guideline for Good Clinical Practice, applicable local regulations, and ethical principles of the Declaration of Helsinki. In addition, the trial was conducted in accordance with US FDA 21 Code of Federal Regulations parts 50, 56, and 312 and the Clinical trials–Directive 2001/20/EC of the European Parliament and of the Council. All patients provided signed, informed consent before any trial-related procedures were carried out. The manuscript was drafted following the relevant portions of the CONSORT guidelines and checklist, as this was a phase 1/2 open-label nonrandomized study.

The sponsor discontinued the clinical development of EnaV in November 2020 but continued to offer treatment to those who were still deriving clinical benefit. Dose-escalation and dose-expansion data collected up to and including the data cutoff of March 22, 2021, are included in this analysis.

### Patients

In both the dose-escalation and dose-expansion phases, eligible patients were ≥18 years of age, had measurable disease according to RECIST v1.1, Eastern Cooperative Oncology Group performance status of 0 or 1, and a life expectancy of ≥3 months. Key inclusion and exclusion criteria for both the dose-escalation and dose-expansion phases are shown in Supplementary Table S1. Of note, tumoral AXL expression was not assessed prospectively and did not serve as a selection criterion. For the dose-escalation phase, eligible patients had relapsed or refractory NSCLC, melanoma, or ovarian, cervical, endometrial, or thyroid cancer and had disease progression following available standard therapy (or were not candidates for standard therapy). For the dose-expansion phase, eligible patients had advanced and/or metastatic solid tumors, including NSCLC, melanoma, sarcoma, ovarian cancer, and other solid tumors, whose disease had progressed following prior therapies, and were not candidates for standard therapy. Detailed entry criteria for the eight dose-expansion cohorts are summarized in Table 1. Fresh formalin-fixed paraffin-embedded tumor tissue samples were required at screening after failure/stopping of the last prior treatment for patients in the dose-expansion phase; an archival tumor sample was acceptable for the dose-escalation part. Patients were excluded if they had acute deep vein thrombosis (or clinically relevant pulmonary embolism) not stable for at least 4 weeks prior to the first dose of EnaV, history of thromboembolic events, clinically significant cardiac disease, ongoing or recent significant autoimmune disease, history of grade ≥3 immune-related adverse events (AE), or active grade ≥2 peripheral neuropathy. Patients who received major surgery within 4 weeks before the first dose of EnaV were excluded. Patients who received any prior therapy with a conjugated or unconjugated auristatin derivative/vinca-binding site targeting payload were excluded.

### Endpoints and assessments

The primary objective was to determine the MTD (dose escalation) and safety profile of EnaV in patients with select solid tumors (dose expansion). Secondary objectives included characterization of EnaV PK, evaluation of the antitumor activity (measured by tumor shrinkage and per RECIST v1.1), and retrospective evaluation of AXL expression in tumor biopsies. The primary endpoints were DLTs and the severity and frequency of AEs per NCI Common Toxicity Criteria for Adverse Events v4.03. The secondary endpoints included safety laboratory parameters, PK profile, immunogenicity, antitumor activity, and AXL expression in tumor biopsy. Efficacy was assessed by tumor imaging via CT scan/objective response based on RECIST v1.1, overall survival (OS), progression-free survival (PFS), and duration of response. CT imaging was performed at baseline and every 6 weeks [in cohorts treated on Q3W schedule and cohort 8 (3Q4W schedule)] or every 8 weeks [in cohort 6 (3Q4W schedule)] ± 7 days from cycle 1 day 1 until disease progression per RECIST v1.1. Efficacy assessments were performed by the investigator. In addition, assessments of CA-125 response (19) were performed for any patient with ovarian cancer.

Safety assessments, performed throughout the study and until 30 days after the last study treatment, included the frequency and severity of treatment-emergent AEs (TEAEs), laboratory assessments, vital signs, electrocardiograms, and physical examinations. TEAEs were coded using Medical Dictionary for Regulatory Activities (MedDRA) v24.0, and TEAEs and laboratory assessments were graded using NCI Common Toxicity Criteria for Adverse Events v4.03. AEs of special interest (AESIs) were selected based on the events previously reported for other vedotin ADCs and included constipation, neutropenia, peripheral neuropathy, and immune-related AEs (11–13, 20).

AXL expression in the tumor biopsies collected at screening was measured at a central laboratory, using an IHC assay (antibody clone 7E10, 1:10,000; Thermo Fisher Scientific; RRID: AB_10978746) on an automated staining platform. Tumor sections were scored for AXL expression by a certified pathologist both in terms of percentage of tumor cells staining positive for AXL and AXL staining intensity across three levels (“1+ = weak,” “2+ = moderate,” and “3+ = strong”) as previously described in the literature (21). AXL expression was reported as both the percentage of tumor cells staining positive for AXL at any intensity (≥1+) and the total H-score, which was calculated as the sum of weighted percentiles of tumor cells positive for AXL staining at each intensity level. Blood samples for PK assessment of EnaV and free MMAE were collected before treatment and at intervals after dosing for both treatment schedules in the dose-escalation phase. Two separate assays were used for EnaV, one for detecting EnaV only, and one for detecting EnaV and nonconjugated HuMax-AXL.

### Statistical analysis

All patients who received at least one dose of EnaV were included in both the safety and efficacy analyses. Patients who received at least one dose of study drug and had at least one postdose PK measurement were included in the PK analysis set. Data were summarized using descriptive statistics for continuous data and/or contingency tables for categorical data.

### Exploratory analysis

A subset of CT images from patients with NSCLC treated with 1.0 mg/kg (3Q4W), 1.8 mg/kg (Q3W), or 2.2 mg/kg EnaV (Q3W) obtained at pretreatment (*n* = 77) and cycle 2 posttreatment (*n* = 50) time points were analyzed. A semiautomatic tumor segmentation was performed by a board-certified, thoracic fellowship–trained radiologist using the ITK-SNAP software (RRID: SCR_017341) (22). All visible tumor tissue identified on the soft-tissue CT window was used as the segmentation target. Tumors were examined using CaPTk (v1.7.2) (23), deriving 225 radiomic features from pretreatment and cycle 2 posttreatment CT images. Batch effects were balanced using nested ComBat (24). Radiomic feature dimensionality was reduced in two stages, all in the absence of clinical outcome information: clustering features with ≥80% similarity and selection of representative features from each cluster followed by principal component (PC) analysis. This approach resulted in reductions to 21 features at pretreatment, 22 features at cycle 2, and 15 longitudinal features (intersection between the 21 and 22 features). Radiomic PC1 was utilized in statistical models to assess OS and PFS with a Cox proportional hazards model, in which PC1 was stratified as high or low risk at the median value or treated as a continuous variable. Overall response rate (ORR) and disease control rate (DCR) were analyzed using a logistic regression model.

## Results

### Patients and treatment exposure

In the dose-escalation phase, 47 patients were enrolled and received treatment: 22 patients (46.8%) had ovarian cancer, 9 (19.1%) had melanoma, 8 (17.0%) had NSCLC, 5 (10.6%) had endometrial cancer, and 3 (6.4%) had cervical cancer. Thirty-two patients received EnaV Q3W, and 15 received EnaV 3Q4W (Supplementary Fig. S1). In the dose-expansion phase, 189 patients received the Q3W schedule with a median of 2 (range, 1–30) doses of EnaV, and 70 patients received the 3Q4W schedule with a median of 6 (range, 1–33) doses of EnaV. At the time of data cutoff, 256 of 259 patients had discontinued treatment; the most common reason for discontinuation was disease progression (64.6% and 71.4% in the Q3W and 3Q4W dose schedule groups, respectively).

Demographic and baseline characteristics of patients in the eight cohorts included in the dose-expansion phase are shown in Table 2. Patients included in this study were generally representative of patients with advanced solid tumors across broader populations in clinical practice (Supplementary Table S2). Patients in the expansion cohorts received a median of 1 to ≥3 prior lines of systemic anticancer therapy; the most heavily pretreated cohorts were cohort 1 (NSCLC) and cohort 7 (ovarian cancer), with 63.6% and 52.0% of patients receiving ≥3 lines of prior therapy, respectively. Among patients with evaluable tumor tissue, median AXL expression (percentage of AXL-positive tumor cells) at baseline ranged from 1.0% to 5.5% in the NSCLC cohorts and 10.0% to 15.0% in the melanoma cohorts; this was 68.8% in the sarcoma cohort, 1.0% in the mixed solid tumors cohort that received prior PD-1/PD-L1 inhibitor treatment, and 5.0% in the ovarian cancer cohort.

**Table 2.** Demographics and baseline characteristics of patients in the dose-expansion population by cohort.

Cohort	NSCLC				Melanoma		Sarcoma	Solid tumors	Ovarian cancer
	1	2		8	3	4	5	6	7
	2.2 mg/kg Q3W (*n* = 22)	2.2 mg/kg Q3W (*n* = 55)	1.8 mg/kg Q3W (*n* = 21)	1.0 mg/kg 3Q4W (*n* = 26)	2.2 mg/kg Q3W (*n* = 16)	2.2 mg/kg Q3W (*n* = 25)	2.2 mg/kg Q3W (*n* = 25)	1.0 mg/kg 3Q4W (*n* = 44)	2.2 mg/kg Q3W (*n* = 25)
Female, *n* (%)	13 (59.1)	25 (45.5)	11 (52.4)	13 (50.0)	8 (50.0)	7 (28.0)	14 (56.0)	17 (38.6)	25 (100.0)
Median age (range, years)	64.0 (48–75)	66.0 (38–77)	61.0 (37–78)	59.5 (39–77)	58.0 (38–78)	64.0 (35–79)	58.0 (36–81)	63.5 (23–80)	62.0 (41–80)
≥65 years of age, %	45.5	56.4	28.6	34.6	12.5	48.0	40.0	47.7	36.0
White race, *n* (%)	18 (81.8)	50 (90.9)	20 (95.2)	23 (88.5)	12 (75.0)	21 (84.0)	24 (96.0)	37 (84.1)	20 (80.0)
ECOG PS, *n* (%)	​	​	​	​	​	​	​	​	​
0	4 (18.2)	13 (23.6)	4 (19.0)	6 (23.1)	7 (43.8)	13 (52.0)	8 (32.0)	11 (25.0)	7 (28.0)
1	17 (77.3)	42 (76.4)	17 (81.0)	20 (76.9)	9 (56.3)	12 (48.0)	17 (68.0)	33 (75.0)	18 (72.0)
2	1 (4.5)	—	—	—	—	—	—	—	—
Median % AXL-positive tumor cells (range) (*n*)	5.5 (0–73) (*n* = 18)	2 (0–90) (*n* = 34)	1 (0–60) (*n* = 15)	5 (0–98) (*n* = 22)	10 (0–95) (*n* = 12)	15 (0–100) (*n* = 19)	68.8 (0–95) (*n* = 14)	1 (1–100) (*n* = 34)	5 (0–80) (*n* = 16)
Cancer type, *n* (%)	​	​	​	​	​	​	​	​	​
NSCLC	22 (100.0)	55 (100.0)	21 (100.0)	26 (100.0)	0	0	0	0	0
Melanoma	0	0	0	0	16 (100.0)	25 (100.0)	0	0	0
Sarcoma	0	0	0	0	0	0	25 (100.0)	0	0
Ovarian cancer	0	0	0	0	0	0	0	0	25 (100.0)
Colorectal cancer	0	0	0	0	0	0	0	7 (15.9)	0
HNSCC	0	0	0	0	0	0	0	7 (15.9)	0
Mesothelioma	0	0	0	0	0	0	0	6 (13.6)	0
RCC	0	0	0	0	0	0	0	5 (11.4)	0
Miscellaneous cancers	0	0	0	0	0	0	0	19 (43.2)	0
Median no. prior systemic anticancer therapy lines (range)	3 (0–4)	2 (0–4)	1 (1–2)	2 (0–3)	2 (0–3)	2 (0–3)	1 (0–3)	2 (0–7)	3 (0–5)

Abbreviations: ECOG, Eastern Cooperative Oncology Group; HNSCC, head and neck squamous cell carcinoma; PS, performance status; RCC, renal cell carcinoma.

### Safety

#### Dose-escalation phase: determination of MTD

Patients received a median of 4 (range, 1–20) and 6 (range, 2–18) doses of EnaV in the Q3W and 3Q4W dosing schedules, respectively. All patients in the dose-escalation phase experienced TEAEs; the incidence of treatment-related AEs (TRAEs) was 96.9% in the EnaV Q3W and 100.0% in the EnaV 3Q4W groups. A total of four DLTs were observed in the Q3W group at doses of 2.0 mg/kg (grade 3 constipation), 2.2 mg/kg (grade 3 constipation and grade 3 vomiting), and 2.4 mg/kg (grade 3 gamma-glutamyl transferase increase). Two DLTs were observed in the 3Q4W group, both at the 1.2-mg/kg dose (febrile neutropenia and diarrhea). The MTD and RP2D were determined to be 2.2 mg/kg for the Q3W dose schedule and 1.0 mg/kg for the 3Q4W dose schedule; per protocol, some patients in cohort 2 of the dose-expansion phase received an EnaV dose of 1.8 mg kg Q3W. A summary of safety and TEAEs reported in ≥20% of patients in the EnaV Q3W and 3Q4W groups are shown in Supplementary Tables S3 and S4.

#### Dose-expansion phase

TEAEs observed in ≥20% of patients in expansion cohorts are shown in Supplementary Table S5. Grade 3 or 4 TEAEs were observed in 65.6% (*n* = 124) of patients in the EnaV Q3W group and 48.6% (*n* = 34) of patients in the 3Q4W group. The most frequent TRAEs reported in the EnaV Q3W and 3Q4W groups were fatigue (48.1%; 42.9%), constipation (47.1%; 30.0%), nausea (40.7%; 28.6%), and decreased appetite (26.5%; 27.1%), followed by alopecia (24.9%) in the Q3W group and peripheral sensory neuropathy (24.3%) in the 3Q4W group (Table 3).

**Table 3.** TRAEs (reported as PTs) in ≥20% of patients, grade ≥3 TRAEs in ≥5% of patients, and AESIs in ≥10% of patients in the dose-expansion phase.

TRAE, *n* (%)	EnaV Q3W (*n* = 189)		EnaV 3Q4W (*n* = 70)	
	All AEs	Grade ≥3	All AEs	Grade ≥3
Fatigue	91 (48.1)	7 (3.7)	30 (42.9)	3 (4.3)
Constipation	89 (47.1)	14 (7.4)	21 (30.0)	3 (4.3)
Nausea	77 (40.7)	3 (1.6)	20 (28.6)	0
Decreased appetite	50 (26.5)	1 (0.5)	19 (27.1)	0
Alopecia	47 (24.9)	—	7 (10.0)	—
Vomiting	34 (18.0)	2 (1.1)	9 (12.9)	0
Diarrhea	35 (18.5)	5 (2.6)	10 (14.3)	0
Peripheral sensory neuropathy	34 (18.0)	3 (1.6)	17 (24.3)	1 (1.4)
Abdominal pain	30 (15.9)	2 (1.1)	2 (2.9)	0
Neutropenia	36 (19.0)	24 (12.7)	2 (2.9)	1 (1.4)
AST increased	30 (15.9)	4 (2.1)	2 (2.9)	0
ALT increased	29 (15.3)	3 (1.6)	3 (4.3)	0
Hyponatremia	11 (5.8)	10 (5.3)	4 (5.7)	4 (5.7)

AESI, *n* (%)	EnaV Q3W (*n* = 189)		EnaV 3Q4W (*n* = 70)	
AESI in ≥10%	All grades	Grade ≥3	All AEs	Grade ≥3
Constipation	89 (47.1)	15 (7.9)	21 (30.0)	3 (4.3)
Neutropenia	36 (19.0)	27 (14.3)	2 (2.9)	1 (1.4)
Diarrhea	35 (18.5)	6 (3.2)	10 (14.3)	0
Vomiting	34 (18.0)	5 (2.6)	9 (12.9)	0
Peripheral sensory neuropathy	34 (18.0)	1 (0.5)	17 (24.3)	1 (1.4)
AST increased	30 (15.9)	5 (2.6)	2 (2.9)	1 (1.4)
ALT increased	29 (15.3)	4 (2.1)	3 (4.3)	0
Anemia	25 (13.2)	9 (4.8)	5 (7.1)	1 (1.4)
Peripheral neuropathy	3 (1.6)	0	8 (11.4)	0

Abbreviations: ALT, alanine aminotransferase; AST, aspartate aminotransferase; PT, preferred term.

In the dose expansion phase, a total of 49 (25.9%) patients in the Q3W group and 14 (20.0%) patients in the 3Q4W group experienced a TEAE that led to study drug discontinuation. The most frequent TEAEs leading to discontinuation in the Q3W group were peripheral sensorimotor neuropathy and peripheral sensory neuropathy [7 (3.7%) patients each]. In the 3Q4W group, the most frequent TEAEs leading to discontinuation were peripheral sensorimotor neuropathy [3 (4.3%) patients] and fatigue [2 (2.9%) patients].

The most common AESIs reported in the EnaV Q3W and 3Q4W groups are shown in Table 3. Two patients died because of septic shock considered related to EnaV. Infusion-related reactions were reported in six and two patients in the Q3W and 3Q4W groups, respectively. No safety concerns with respect to changes in laboratory values were observed in any EnaV cohort.

### Efficacy

#### Dose-escalation phase

In patients who received the EnaV Q3W dose schedule (*n* = 32), the ORR was 9.4%, comprising three partial responses, one each at the EnaV 1.5-mg/kg, 2.2-mg/kg, and 2.4-mg/kg dose levels; the DCR consisting of complete response, partial response, and stable disease (SD) was 68.8%. No responses were observed in patients who received the EnaV 3Q4W dose schedule (*n* = 15), and the DCR was 46.7%.

#### Dose-expansion phase

**Figure 1.**
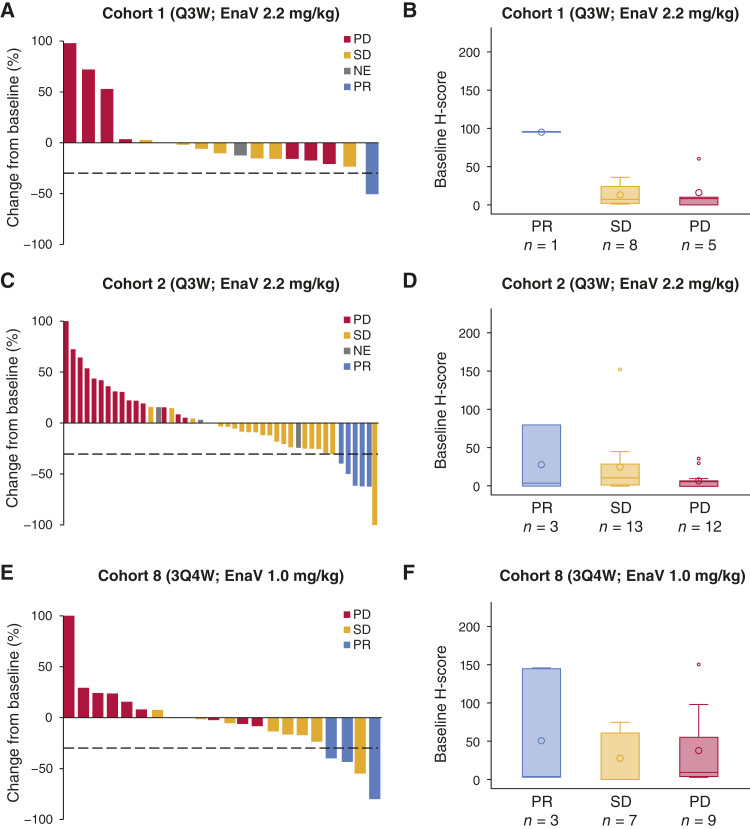
Best change from baseline in sum of target lesions (investigator assessment) in the dose-expansion phase for the NSCLC cohorts (**A**, **C**, and **E**) and baseline tumor H-score levels for AXL positivity by investigator-assessed confirmed best overall response (**B**, **D**, and **F**). **A** and **B,** Cohort 1 (Q3W): NSCLC with sensitizing *EGFR* mutations and/or mutations targeted by third-generation TKIs (*n* = 22). **C** and **D,** Cohort 2 (2.2 mg/kg Q3W): NSCLC without activating *EGFR* mutations or *ALK* rearrangements (*n* = 55). **E** and **F,** Cohort 8 (3Q4W): NSCLC without activating *EGFR* mutations or *ALK* rearrangements (*n* = 26). NE, not evaluable; PD, progressive disease; PR, partial response.

Efficacy outcomes for the dose-expansion cohorts are shown in Table 4. Overall, the investigator-assessed ORR per RECIST ranged from 4.5% to 12.5% across all Q3W dose schedule cohorts and from 9.1% to 11.5% across all 3Q4W dose schedule cohorts. Among patients with NSCLC who received the EnaV Q3W dose schedule [cohorts 1 (*n* = 22) and 2 (*n* = 76)], the ORR per investigator assessment varied from 4.5% to 9.1%, DCR varied from 47.6% to 49.1%, median PFS varied from 2.2 to 2.6 months, and median OS varied from 8.7 to 16.5 months. Among patients with NSCLC who received the EnaV 3Q4W dose schedule [cohort 8 (*n* = 26)], the investigator-assessed ORR was 11.5% (95% confidence interval, 2.4%–30.2%), DCR was 50.0%, median PFS was 2.0 months, and median OS was 11.4 months. Waterfall plots showing best change from baseline in sum of target lesions for the NSCLC dose-expansion cohorts, along with AXL expression in relation to efficacy outcomes, are shown in Fig. 1. Among the other cohorts, responses were observed in patients with melanoma (ORR, 12.0%–12.5%), ovarian cancer (ORR, 8.0%), and solid tumors (ORR, 9.1%). In the Q3W ovarian cancer cohort, 9/15 (60%) patients were evaluable for CA-125; one patient in the 2.4 mg/kg cohort had a CA-125 response. In the 3Q4W ovarian cancer cohort, 5/7 (71.4%) patients were evaluable for CA-125; no CA-125 responses were observed.

**Table 4.** Clinical activity in the dose-expansion phase (confirmed responses per investigator assessment).

Cohort	NSCLC				Melanoma		Sarcoma	Solid tumor^a^	Ovarian cancer
	1	2		8	3	4	5	6	7
Dose	2.2 mg/kg Q3W (*n* = 22)	2.2 mg/kg Q3W (*n* = 55)	1.8 mg/kg Q3W (*n* = 21)	1.0 mg/kg 3Q4W (*n* = 26)	2.2 mg/kg Q3W (*n* = 16)	2.2 mg/kg Q3W (*n* = 25)	2.2 mg/kg Q3W (*n* = 25)	1.0 mg/kg 3Q4W (*n* = 44)	2.2 mg/kg Q3W (*n* = 25)
Confirmed best overall response, *n* (%)^b^	​	​	​	​	​	​	​	​	​
CR	0	0	0	0	0	0	0	1 (2.3)	0
PR	1 (4.5)	5 (9.1)	1 (4.8)	3 (11.5)	2 (12.5)	3 (12.0)	0	3 (6.8)	2 (8.0)
SD	8 (36.4)	22 (40.0)	9 (42.9)	10 (38.5)	7 (43.8)	14 (56.0)	15 (60.0)	12 (27.3)	10 (40.0)
PD	8 (36.4)	16 (29.1)	7 (33.3)	10 (38.5)	5 (31.3)	7 (28.0)	5 (20.0)	23 (52.3)	10 (40.0)
NE	5 (22.7)	12 (21.8)	4 (19.0)	3 (11.5)	2 (12.5)	1 (4.0)	5 (20.0)	5 (11.4)	3 (12.0)
ORR, % (95% CI)	4.5 (0.1–22.8)	9.1 (3.0–20.0)	4.8 (0.1–23.8)	11.5 (2.4–30.2)	12.5 (1.6–38.3)	12.0 (2.5–31.2)	0	9.1 (2.5–21.7)	8.0 (1.0–26.0)
DCR, % (95% CI)	40.9 (20.7–63.6)	49.1 (35.4–62.9)	47.6 (25.7–70.2)	50.0 (29.9–70.1)	56.3 (29.9–80.2)	68.0 (46.5–85.1)	60.0 (38.7–78.9)	36.4 (22.4–52.2)	48.0 (27.8–68.7)
Median PFS, months (95% CI)	2.6 (1.2–2.8)	2.2 (1.4–3.9)	2.6 (1.1–4.0)	2.0 (1.4–4.1)	2.6 (1.2–3.9)	2.8 (1.3–5.0)	2.6 (1.4–4.1)	1.9 (1.6–2.1)	1.6 (1.3–3.0)
Median time to response, months (95% CI)	1.2 (NR–NR)	1.6 (1.2–NR)	1.4 (NR–NR)	1.6 (1.3–NR)	2.8 (2.5–NR)	1.7 (1.6–NR)	—	1.6 (1.5–NR)	1.2 (1.2–NR)
Median OS, months (95% CI)	16.5 (4.1–NR)	11.3 (8.0–15.5)	8.7 (5.8–14.1)	11.4 (4.1–NR)	8.6 (3.6–19.1)	9.2 (4.0–14.9)	17.1 (5.7–24.3)	7.0 (5.2–NR)	8.4 (6.5–12.4)

Abbreviations: CI, confidence interval; CR, complete response; DCR, disease control rate (CR + PR + SD); NE, not evaluable; NR, not reached; PD, progressive disease; PR, partial response.

a Includes colorectal cancer (*n* = 7), mesothelioma (*n* = 6), renal cell carcinoma (*n* = 5), squamous cell carcinoma of the head and neck (*n* = 7), and other miscellaneous cancers (*n* = 19) excluding NSCLC, melanoma, sarcoma, and ovarian cancer unless a known *AXL* gene amplification was present.

b Assessed by investigator.

Box-and-whisker plots showing baseline tumor H-score levels for AXL expression by best overall response by investigator assessment for the other cohorts are shown in Supplementary Fig. S2, with no consistent differences observed across clinical response categories.

### PK

**Figure 2.**
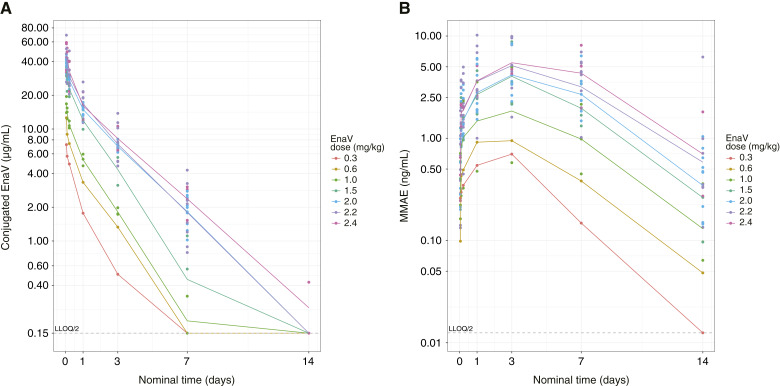
PK profile for EnaV and MMAE in the dose-escalation phase. Plasma/serum concentrations of (**A**) conjugated EnaV and (**B**) MMAE at Q3W. Dots indicate observed data; lines indicate mean data. LLOQ, lower limit of quantitation.

PK data from patients treated with EnaV 0.3 to 2.4 mg/kg Q3W and with EnaV 0.6 to 1.2 mg/kg 3Q4W in the dose-escalation phase indicate that there was dose-related increase for both EnaV and MMAE, as shown in Fig. 2 and Supplementary Fig. S3. EnaV showed a relatively fast elimination, with median half-life ranging from 0.9 to 2.2 days (Q3W) and 1.1 to 2.1 days (3Q4W) across the dose range tested. The C_max_ for EnaV was observed shortly after infusion, and the MMAE concentrations peaked around day 4 (Fig. 2).

#### Exploratory image-based analysis

The cycle 2 radiomics signature and the longitudinal signature both correlated with disease control [both *P* = 0.03; OR: 1.37 (95% confidence interval, 1.02–1.82); [Supplementary Table S6]]. We observed that radiomics features capturing the change in tumor characteristics over the treatment course differed from those that identified variability in tumor structure at static time points (Supplementary Table S7). For example, unlike the baseline and cycle 2 radiomics signatures, the imaging features contributing most to the longitudinal radiomics signature did not include shape features. No association between radiomics signatures and OS or PFS was observed.

## Discussion

This first-in-human, phase 1/2, dose-escalation and dose-expansion study sought to define the MTD and RP2D and determine the efficacy and safety of EnaV, an AXL-directed ADC, in a mixed population of patients with solid tumors, including NSCLC, melanoma, ovarian cancer, sarcoma, and other malignancies. The dose-expansion phase was designed to provide further data on the safety, tolerability, and antitumor activity of the MTD/RP2D of EnaV from the dose-escalation phase: 2.2/1.8 mg/kg with the Q3W dosing schedule and 1.0 mg/kg with the 3Q4W dosing schedule. The populations included in this study were selected based on known or hypothesized high expression of tumoral AXL supported by reports in the literature that tumoral AXL expression is increased upon development of drug resistance in prior lines of therapy (1, 3–8). Patients with NSCLC were the major focus of this dose-optimization study as initial studies showed promise in this patient population, and patients were stratified by *EGFR* mutation status based on the potential role of AXL overexpression in acquired resistance to TKI therapy (5). The inclusion of platinum-resistant ovarian cancer as an expansion cohort in the study was based on the literature suggesting that platinum resistance may promote a mesenchymal phenotype, which is associated with elevated AXL expression in tumors (25). The sarcoma cohort was included based on the known intrinsically high expression of AXL in these tumors as well as preclinical studies that showed EnaV was able to elicit antitumor activity in patient-derived xenograft models of soft-tissue sarcoma (17). In contrast, pancreatic cancer was excluded because of its presumed intrinsic resistance to antitubulin agents and its high expression of ATP-binding cassette drug transporters, including MDR1 (26, 27).

AXL expression is dynamic, and patients were not assessed for AXL expression in their tumors by IHC prior to study enrollment. To enable prospective patient selection based on AXL expression, a validated IHC assay with a clinically defined expression cutoff is required. However, as this was a first-in-human study with EnaV, the threshold of tumoral AXL expression needed to elicit clinical activity was unknown. Moreover, it was hypothesized that AXL-low or AXL-negative tumors might still benefit from EnaV through bystander effects mediated by AXL-expressing immune cells. For these reasons, tumor AXL expression was assessed retrospectively to determine its potential as an enrichment biomarker. Retrospective analysis of AXL expression shown in Fig. 1 and Supplementary Fig. S2 highlights the variability observed among patient samples in this study.

In the dose-expansion phase, EnaV showed a safety profile consistent with that expected for an ADC with an MMAE payload, including AESIs of constipation, neutropenia, and peripheral neuropathy. AESIs were generally comparable with what was observed with other ADCs with a similar payload, although rates of constipation were higher among EnaV-treated patients (11–14). The most common TRAEs in this study were fatigue (48.1%), constipation (47.1%), and nausea (40.7%) in the EnaV Q3W group and fatigue (42.9%), constipation (30.0%), nausea (28.6%), and decreased appetite (27.1%) in the EnaV 3Q4W group. Overall, patients in the EnaV Q3W group (*n* = 189) had a higher incidence of serious AEs (51.9% vs. 42.9%, respectively) and AEs leading to discontinuation (25.9% vs. 20.0%) compared with the 3Q4W group (*n* = 70). These discontinuation rates were similar to those observed for other ADCs with a similar payload (11–13, 20). Two patients experienced fatal TEAEs considered related to EnaV treatment; both deaths were due to septic shock and occurred during the first treatment cycle in patients receiving EnaV 2.2 mg/kg Q3W. It is important to note that late-stage cancer is a risk factor for developing sepsis (28), and rates of serious neutropenia and febrile neutropenia in our study were low, occurring in 3 (1.2%) patients and 4 (1.5%) patients, respectively.

EnaV treatment both at 2.2 mg/kg Q3W and 1.0 mg/kg 3Q4W did not result in clinically meaningful responses in our unselected population of patients with recurrent or metastatic cancers who had recent disease progression on or after chemotherapy. Of note, the results showed that the ORRs with EnaV were not higher than those reported for most single-agent chemotherapy options used currently in therapy for similarly eligible patients. Among patients with advanced/recurrent NSCLC, response rates to chemotherapy in the second-line setting historically range from 9% to 17% (29–33), which is comparable with the response rates observed in patients with NSCLC in the current study. Most patients with NSCLC showed reduction in tumor size upon receiving EnaV; however, many of these reductions in tumor size were not sufficient for patients to be classified as having a response. Patients with NSCLC receiving EnaV Q3W showed similar DCRs to those receiving EnaV 3Q4W (40.9%–49.1% vs. 50.0%, respectively). Due to factors such as low response rates, interpatient variability, and available samples in the biomarker-evaluable population, radiomics-based exploratory analyses did not produce conclusive results. Dose optimization (Q3W and 3Q4W) did not improve the risk/benefit ratio sufficiently to justify further development for monotherapy use, and the sponsor ceased further clinical development.

Tumoral AXL expression at baseline (from tumor biopsies collected following disease progression on the last prior therapy) was generally low (≤15% median AXL+ tumor cells) in all cohorts except for sarcoma (68.8% median AXL+ tumor cells) (Table 2), a tumor type with known high intrinsic AXL expression. These findings do not support the original hypothesis that AXL expression is induced as a robust resistance mechanism to prior therapy. Additionally, tumoral AXL expression by IHC showed a high degree of heterogeneity among partial responders and was not significantly associated with clinical response (Fig. 1; Supplementary Fig. S2).

Whereas the low levels of AXL expression in some of the cohorts may explain the lack of clinically meaningful activity by EnaV, no objective responses were observed in patients in the sarcoma cohort despite having the highest median percentage of AXL-positive tumor cells. The relative lack of objective response in the sarcoma cohort is likely due to the aggressive nature of the disease and intrinsic resistance of the tumor cells to tubulin-targeting payloads, including MMAE (34), that could not be overcome despite the increased payload delivery facilitated by high tumoral AXL expression. However, because sarcoma is heterogenous and frequently does not respond to treatment, SD is often considered a clinically meaningful outcome in this indication. Indeed, patients with sarcoma had a high rate of SD (60%) and the longest median OS (17.1 months) of any tumor type included in this study. Other ADCs, including trastuzumab deruxtecan and sacituzumab govitecan, have shown efficacy despite the low expression of antibody target (35, 36). Nonetheless, this result raises questions about the potential for EnaV in AXL-expressing tumors. A potential limitation of our study is the lack of on-treatment biopsy data confirming MMAE delivery to the tumor, which prevented the investigation of drug delivery. Without a biomarker to confirm whether the MMAE is delivered to the tumor site, payload delivery remains uncharacterized, potentially confounding strategies to increase efficacy. The efficacy observed with ADCs containing MMAE as a payload suggests that impaired payload delivery could negatively affect clinical activity. However, it is possible that the tumor types in studies of other MMAE-containing ADCs may be more sensitive to the payload (11–14). It is also possible that other factors, including internalization and/or intracellular release of the cytotoxic payload, as well as a payload resistance mechanism (e.g., drug efflux pumps), could contribute to the lack of clinically meaningful activity even in AXL-positive tumors. Increasing the payload-to-antibody ratio could potentially mitigate these mechanisms and increase efficacy, particularly among patients with NSCLC, as free MMAE can generate bystander toxicity to eliminate surrounding tumor cells (15).

Previous studies have reported an association between AXL expression and drug resistance (4, 5, 9). One possible mechanism is cross-talk between AXL and other RTK family members. AXL is able to heterodimerize with TAM/non-TAM RTK family members, such as EGFR, MET, and platelet-derived growth factor, allowing them to avoid the effects of TKIs (37). For example, heterointeractions between AXL and *HER2* and AXL and *EGFR* have been shown to activate downstream signaling pathways. Therefore, it is possible that AXL-targeted therapy as a component of combination treatment may be able to prevent escape due to reverse cross-talk, as opposed to single-agent therapy. In preclinical studies using a melanoma model, the activity of an AXL-directed ADC was potentiated by combination with MAPK inhibitors (15), which also suggests a potential role for AXL-directed therapy in combination with other targeted therapies. Another possible approach to improving activity in future studies is to use an alternative chemotherapeutic component. A recent preclinical study with an AXL-targeted ADC conjugated to a pyrrolobenzodiazepine cytotoxin showed potentially promising results in a *BRCA1-*mutated ovarian cancer model, highlighting the potential benefit of ADCs with varied mechanisms of action (38).

Retrospective exploratory analysis of CT images from a subset of patients with NSCLC demonstrated a correlation between disease control and radiomics signatures at both cycle 2 and longitudinally. Though preliminary, these results are notable given that the signatures were derived independent of any clinical outcome and among different dose levels and schedules. The hypotheses behind this analysis are linked to radiomics features not visually apparent using standard imaging approaches. These features capture characteristics of tumor heterogeneity typically associated with intratumoral genetic or molecular variability, the tumor-immune microenvironment composition, or vascular biology—all of which have been reported to predict clinical outcomes in patients treated with different drugs (39–43). Notably, the primary contributors to the longitudinal radiomics signature excluded any shape features included in the baseline and cycle 2 radiomics signature, suggesting that the change in overall tumor form may not be the most important indicator of longitudinal response in this cohort. Further validation of these signatures in an independent NSCLC patient cohort is required to assess the robustness of their prognostic and predictive effects.

In addition to its role as a transforming oncogene, AXL has emerged as a promising target for immuno-oncology because of its involvement in modulating innate immune responses and immune surveillance within the tumor microenvironment (44). Multiple compounds targeting AXL in breast cancer are currently under development, particularly in combination with immune checkpoint inhibitors (45). To the best of our knowledge, combining an AXL-targeted ADC with an immune checkpoint inhibitor has not been clinically tested but may hold potential for further development in the field of immuno-oncology. Clinical trials are currently underway to determine the safety and preliminary efficacy of chimeric antigen receptor (CAR)-engineered immune cells, including a phase 1 study of CAR T-cell therapy in patients with advanced AXL-positive lung cancer (NCT03198052) and a phase 1 trial of CAR-NK cells in AXL-positive ovarian cancer and other solid tumors (NCT05410717). Other types of AXL-targeted therapies undergoing clinical development include small-molecule inhibitors, anti-AXL mAbs, and AXL-targeting ADCs. Highly selective small-molecule inhibitors, such as S49076 and bemcentinib (BGB324), have shown preliminary antitumor activity in phase 1 and phase 2 trials, respectively, in patients with advanced solid tumors (46, 47). Tilvestamab, an anti-AXL mAb, demonstrated a tolerable safety profile with limited preliminary antitumor activity in a phase 1 clinical trial of patients with relapsed, platinum-resistant high-grade serous ovarian cancer (48). Mecbotamab vedotin (BA3011) is a conditionally active anti-AXL and MMAE ADC currently under clinical investigation in a phase 1/2 study of mecbotamab vedotin alone or in combination with nivolumab in adult and adolescent patients with advanced refractory sarcoma (NCT03425279) (49, 50). Preliminary results showed an acceptable safety profile and encouraging disease control; among 112 response-evaluable patients in the mecbotamab vedotin monotherapy and mecbotamab vedotin + nivolumab arms combined, 5 (4.5%) treatment responses were observed, with a DCR of 41% and an estimated 12-week PFS rate of 40% (50).

In conclusion, although EnaV showed a manageable safety profile that was consistent with that of other ADCs in the vedotin class, antitumor activity as monotherapy was modest in the selected tumor types. Of note, tumoral AXL expression at baseline was highly heterogeneous among patients with clinical response to EnaV. Further investigations exploring drug delivery to the tumor site, alternative methods, and combination therapies are needed to assess the potential of AXL as a target for the treatment of solid tumors.

## Supplementary Material

Table S1 Key inclusion and exclusion criteria

Table S2 Representativeness of study participants

Table S3 Safety summary: TEAEs in ≥5% of patients overall and grade ≥3 TEAEs in dose-escalation phase (Q3W schedule)

Table S4 Safety summary: TEAEs in ≥5% of patients overall in dose-escalation phase (3Q4W schedule)

Table S5 Safety summary: TEAEs in ≥20% of patients in any cohort in dose-expansion phase

Table S6 Coefficient p-values for logistic regression models of investigator confirmed ORR and DCR built from radiomic feature-derived signatur

Table S7 Loading values of radiomic features for principal component 1 comprising radiomics signatures at baseline, cycle 2, and longitudinal; the top 5 contributors to each radiomics signature highlighted with colored arrows

Figure S1 Design of dose-escalation phase. CRM, continuous reassessment model; Q3W, once every 3 weeks; 3Q4W, 3 weekly doses every 4 weeks

Figure S2 Baseline tumor H-score levels for AXL positivity by best confirmed overall response per investigator assessment in expansion cohorts. (A) Cohort 3 (Q3W): melanoma with BRAF V600 mutation; (B) cohort 4 (Q3W): melanoma with BRAF V600 wild-type; (C) cohort 5 (Q3W): sarcoma; (D) cohort 6 (3Q4W): other metastatic solid tumors; and (E) cohort 7 (Q3W): platinum-resistant ovarian cancer . CR, complete response; PD, progressive disease; PR, partial response; Q3W, once every 3 weeks; 3Q4W, 3 weekly doses every 4 weeks; SD, stable disease

Figure S3 Pharmacokinetic profile for EnaV in dose-escalation phase. Plasma/serum concentrations of (A) conjugated EnaV and (B) MMAE at 3Q4W. Dots indicate observed data; lines indicate mean data. EnaV, enapotamab vedotin; LLOQ, lower limit of quantitation; MMAE, monomethyl auristatin E

## Data Availability

Clinical trial data can be requested by qualified researchers for use in rigorous, independent scientific research as long as the trials are not part of an ongoing or planned regulatory submission. Sharing of data is subject to protection of patient privacy and respect for the patient’s informed consent. The data will be provided following review and approval of a research proposal and Statistical Analysis Plan and execution of a Data Sharing Agreement Data. For approved requests, the data will be accessible for 12 months, with possible extensions considered. For more information on the process or to submit a request, contact clinicaltrials@genmab.com.

## References

[1] Sang YB , Kim J-H, Kim C-G, Hong MH, Kim HR, Cho BC, . The development of AXL inhibitors in lung cancer: recent progress and challenges. Front Oncol 2022;12:811247. 35311091 10.3389/fonc.2022.811247 PMC8927964

[2] Zaman A , Bivona TG. Targeting AXL in NSCLC. Lung Cancer (Auckl) 2021;12:67–79. 34408519 10.2147/LCTT.S305484 PMC8364399

[3] Wang K-H , Ding D-C. Dual targeting of TAM receptors Tyro3, Axl, and MerTK: role in tumors and the tumor immune microenvironment. Tzu Chi Med J 2021;33:250–6. 34386362 10.4103/tcmj.tcmj_129_20 PMC8323642

[4] Byers LA , Diao L, Wang J, Saintigny P, Girard L, Peyton M, . An epithelial-mesenchymal transition gene signature predicts resistance to EGFR and PI3K inhibitors and identifies Axl as a therapeutic target for overcoming EGFR inhibitor resistance. Clin Cancer Res 2013;19:279–90. 23091115 10.1158/1078-0432.CCR-12-1558 PMC3567921

[5] Zhang Z , Lee JC, Lin L, Olivas V, Au V, LaFramboise T, . Activation of the AXL kinase causes resistance to EGFR-targeted therapy in lung cancer. Nat Genet 2012;44:852–60. 22751098 10.1038/ng.2330 PMC3408577

[6] Aguilera TA , Rafat M, Castellini L, Shehade H, Kariolis MS, Hui AB-Y, . Reprogramming the immunological microenvironment through radiation and targeting Axl. Nat Commun 2016;7:13898. 28008921 10.1038/ncomms13898 PMC5196438

[7] Hugo W , Zaretsky JM, Sun L, Song C, Moreno BH, Hu-Lieskovan S, . Genomic and transcriptomic features of response to anti-PD-1 therapy in metastatic melanoma. Cell 2016;165:35–44. 26997480 10.1016/j.cell.2016.02.065 PMC4808437

[8] Wang C , Jin H, Wang N, Fan S, Wang Y, Zhang Y, . Gas6/Axl axis contributes to chemoresistance and metastasis in breast cancer through Akt/GSK-3β/β-catenin signaling. Theranostics 2016;6:1205–19. 27279912 10.7150/thno.15083 PMC4893646

[9] Hu S , Dai H, Li T, Tang Y, Fu W, Yuan Q, . Broad RTK-targeted therapy overcomes molecular heterogeneity-driven resistance to cetuximab via vectored immunoprophylaxis in colorectal cancer. Cancer Lett 2016;382:32–43. 27569653 10.1016/j.canlet.2016.08.022

[10] Fu Z , Li S, Han S, Shi C, Zhang Y. Antibody drug conjugate: the “biological missile” for targeted cancer therapy. Signal Transduct Target Ther 2022;7:93. 35318309 10.1038/s41392-022-00947-7 PMC8941077

[11] Younes A , Gopal AK, Smith SE, Ansell SM, Rosenblatt JD, Savage KJ, . Results of a pivotal phase II study of brentuximab vedotin for patients with relapsed or refractory Hodgkin’s lymphoma. J Clin Oncol 2012;30:2183–9. 22454421 10.1200/JCO.2011.38.0410 PMC3646316

[12] Sehn LH , Herrera AF, Flowers CR, Kamdar MK, McMillan A, Hertzberg M, . Polatuzumab vedotin in relapsed or refractory diffuse large B-cell lymphoma. J Clin Oncol 2020;38:155–65. 31693429 10.1200/JCO.19.00172 PMC7032881

[13] Coleman RL , Lorusso D, Gennigens C, González-Martín A, Randall L, Cibula D, . Efficacy and safety of tisotumab vedotin in previously treated recurrent or metastatic cervical cancer (innovaTV 204/GOG-3023/ENGOT-cx6): a multicentre, open-label, single-arm, phase 2 study. Lancet Oncol 2021;22:609–19. 33845034 10.1016/S1470-2045(21)00056-5

[14] Rosenberg JE , O’Donnell PH, Balar AV, McGregor BA, Heath EI, Yu EY, . Pivotal trial of enfortumab vedotin in urothelial carcinoma after platinum and anti-programmed death 1/programmed death ligand 1 therapy. J Clin Oncol 2019;37:2592–600. 31356140 10.1200/JCO.19.01140 PMC6784850

[15] Boshuizen J , Koopman LA, Krijgsman O, Shahrabi A, van den Heuvel EG, Ligtenberg MA, . Cooperative targeting of melanoma heterogeneity with an AXL antibody-drug conjugate and BRAF/MEK inhibitors. Nat Med 2018;24:203–12. 29334371 10.1038/nm.4472

[16] Koopman LA , Terp MG, Zom GG, Janmaat ML, Jacobsen K, Gresnigt-van den Heuvel E, . Enapotamab vedotin, an AXL-specific antibody-drug conjugate, shows preclinical antitumor activity in non-small cell lung cancer. JCI Insight 2019;4:e128199. 31600169 10.1172/jci.insight.128199 PMC6948776

[17] Van Renterghem B , Wozniak A, Castro PG, Franken P, Pencheva N, Sciot R, . Enapotamab vedotin, an AXL-specific antibody-drug conjugate, demonstrates antitumor efficacy in patient-derived xenograft models of soft tissue sarcoma. Int J Mol Sci 2022;23:7493. 35886842 10.3390/ijms23147493 PMC9322120

[18] Boshuizen J , Pencheva N, Krijgsman O, Altimari DD, Castro PG, de Bruijn B, . Cooperative targeting of immunotherapy-resistant melanoma and lung cancer by an AXL-targeting antibody-drug conjugate and immune checkpoint blockade. Cancer Res 2021;81:1775–87. 33531370 10.1158/0008-5472.CAN-20-0434

[19] Rustin GJS , Quinn M, Thigpen T, du Bois A, Pujade-Lauraine E, Jakobsen A, . Re: new guidelines to evaluate the response to treatment in solid tumors (ovarian cancer). J Natl Cancer Inst 2004;96:487–8. 10.1093/jnci/djh081 15026475

[20] Rosenberg JE , Sridhar SS, Zhang J, Smith DC, Ruether JD, Flaig TW, . Mature results from EV-101: a phase I study of enfortumab vedotin in patients with metastatic urothelial cancer (mUC). J Clin Oncol 2019;37(Suppl 7):377. 10.1200/JCO.19.02044 PMC7106979 32031899

[21] Karachaliou N , Chaib I, Cardona AF, Berenguer J, Bracht JWP, Yang J, . Common co-activation of AXL and CDCP1 in EGFR-mutation-positive non-smallcell lung cancer associated with poor prognosis. EBioMedicine 2018;29:112–27. 29433983 10.1016/j.ebiom.2018.02.001 PMC5925453

[22] Yushkevich PA , Piven J, Hazlett HC, Smith RG, Ho S, Gee JC, . User-guided 3D active contour segmentation of anatomical structures: significantly improved efficiency and reliability. Neuroimage 2006;31:1116–28. 16545965 10.1016/j.neuroimage.2006.01.015

[23] Davatzikos C , Rathore S, Bakas S, Pati S, Bergman M, Kalarot R, . Cancer imaging phenomics toolkit: quantitative imaging analytics for precision diagnostics and predictive modeling of clinical outcome. J Med Imaging (Bellingham) 2018;5:011018. 29340286 10.1117/1.JMI.5.1.011018 PMC5764116

[24] Horng H , Singh A, Yousefi B, Cohen EA, Haghighi B, Katz S, . Generalized ComBat harmonization methods for radiomic features with multi-modal distributions and multiple batch effects. Sci Rep 2022;12:4493. 35296726 10.1038/s41598-022-08412-9 PMC8927332

[25] Antony J , Tan TZ, Kelly Z, Low J, Choolani M, Recchi C, . The GAS6-AXL signaling network is a mesenchymal (Mes) molecular subtype-specific therapeutic target for ovarian cancer. Sci Signal 2016;9:ra97. 27703030 10.1126/scisignal.aaf8175

[26] Albahde MAH , Abdrakhimov B, Li G-Q, Zhou X, Zhou D, Xu H, . The role of microtubules in pancreatic cancer: therapeutic progress. Front Oncol 2021;11:640863. 34094924 10.3389/fonc.2021.640863 PMC8176010

[27] Quiñonero F , Mesas C, Doello K, Cabeza L, Perazzoli G, Jimenez-Luna C, . The challenge of drug resistance in pancreatic ductal adenocarcinoma: a current overview. Cancer Biol Med 2019;16:688–99. 31908888 10.20892/j.issn.2095-3941.2019.0252 PMC6936232

[28] Lindström A-C , Eriksson M, Mårtensson J, Oldner A, Larsson E. Nationwide case-control study of risk factors and outcomes for community-acquired sepsis. Sci Rep 2021;11:15118. 34301988 10.1038/s41598-021-94558-x PMC8302728

[29] Brahmer J , Reckamp KL, Baas P, Crinò L, Eberhardt WEE, Poddubskaya E, . Nivolumab versus docetaxel in advanced squamous-cell non-small-cell lung cancer. N Engl J Med 2015;373:123–35. 26028407 10.1056/NEJMoa1504627 PMC4681400

[30] Borghaei H , Paz-Ares L, Horn L, Spigel DR, Steins M, Ready NE, . Nivolumab versus docetaxel in advanced nonsquamous non-small-cell lung cancer. N Engl J Med 2015;373:1627–39. 26412456 10.1056/NEJMoa1507643 PMC5705936

[31] Rittmeyer A , Barlesi F, Waterkamp D, Park K, Ciardiello F, von Pawel J, . Atezolizumab versus docetaxel in patients with previously treated non-small-cell lung cancer (OAK): a phase 3, open-label, multicentre randomised controlled trial. Lancet 2017;389:255–65. 27979383 10.1016/S0140-6736(16)32517-X PMC6886121

[32] Borghaei H , de Marinis F, Dumoulin D, Reynolds C, Theelen WSME, Percent I, . SAPPHIRE: phase III study of sitravatinib plus nivolumab versus docetaxel in advanced nonsquamous non-small-cell lung cancer. Ann Oncol 2024;35:66–76. 37866811 10.1016/j.annonc.2023.10.004

[33] Neal J , Pavlakis N, Kim S-W, Goto Y, Lim SM, Mountzios G, . CONTACT-01: a randomized phase III trial of atezolizumab + cabozantinib versus docetaxel for metastatic non-small cell lung cancer after a checkpoint inhibitor and chemotherapy. J Clin Oncol 2024;42:2393–403. 38552197 10.1200/JCO.23.02166 PMC11227305

[34] Kyle AH , Huxham LA, Yeoman DM, Minchinton AI. Limited tissue penetration of taxanes: a mechanism for resistance in solid tumors. Clin Cancer Res 2007;13:2804–10. 17473214 10.1158/1078-0432.CCR-06-1941

[35] Modi S , Jacot W, Yamashita T, Sohn J, Vidal M, Tokunaga E, . Trastuzumab deruxtecan in previously treated HER2-low advanced breast cancer. N Engl J Med 2022;387:9–20. 35665782 10.1056/NEJMoa2203690 PMC10561652

[36] Bardia A , Rugo HS, Cortés J, Tolaney SM, Schmid P, Motwani M, . Trop-2 mRNA expression and association with clinical outcomes with sacituzumab govitecan (SG) in patients with HR+/HER2− metastatic breast cancer (mBC): biomarker results from the phase 3 TROPiCS-02 study. J Clin Oncol 2023;41(Suppl 16):1082.

[37] Zhu C , Wei Y, Wei X. AXL receptor tyrosine kinase as a promising anti-cancer approach: functions, molecular mechanisms and clinical applications. Mol Cancer 2019;18:153. 31684958 10.1186/s12943-019-1090-3 PMC6827209

[38] Zammarchi F , Havenith KE, Chivers S, Hogg P, Bertelli F, Tyrer P, . Preclinical development of ADCT-601, a novel pyrrolobenzodiazepine dimer-based antibody-drug conjugate targeting AXL-expressing cancers. Mol Cancer Ther 2022;21:582–93. 35086955 10.1158/1535-7163.MCT-21-0715 PMC9377743

[39] Braman N , Prasanna P, Bera K, Alilou M, Khorrami M, Leo P, . Novel radiomic measurements of tumor-associated vasculature morphology on clinical imaging as a biomarker of treatment response in multiple cancers. Clin Cancer Res 2022;28:4410–24. 35727603 10.1158/1078-0432.CCR-21-4148 PMC9588630

[40] Alilou M , Khorrami M, Prasanna P, Bera K, Gupta A, Viswanathan VS, . A tumor vasculature-based imaging biomarker for predicting response and survival in patients with lung cancer treated with checkpoint inhibitors. Sci Adv 2022;8:eabq4609. 36427313 10.1126/sciadv.abq4609 PMC9699671

[41] Alilou M , Orooji M, Beig N, Prasanna P, Rajiah P, Donatelli C, . Quantitative vessel tortuosity: a potential CT imaging biomarker for distinguishing lung granulomas from adenocarcinomas. Sci Rep 2018;8:15290. 30327507 10.1038/s41598-018-33473-0 PMC6191462

[42] Wang X , Xie T, Luo J, Zhou Z, Yu X, Guo X. Radiomics predicts the prognosis of patients with locally advanced breast cancer by reflecting the heterogeneity of tumor cells and the tumor microenvironment. Breast Cancer Res 2022;24:20. 35292076 10.1186/s13058-022-01516-0 PMC8922933

[43] Su G-H , Xiao Y, You C, Zheng R-C, Zhao S, Sun S-Y, . Radiogenomic-based multiomic analysis reveals imaging intratumor heterogeneity phenotypes and therapeutic targets. Sci Adv 2023;9:eadf0837. 37801493 10.1126/sciadv.adf0837 PMC10558123

[44] Engelsen AST , Lotsberg ML, Abou Khouzam R, Thiery J-P, Lorens JB, Chouaib S, . Dissecting the role of AXL in cancer immune escape and resistance to immune checkpoint inhibition. Front Immunol 2022;13:869676. 35572601 10.3389/fimmu.2022.869676 PMC9092944

[45] Adam-Artigues A , Arenas EJ, Arribas J, Prat A, Cejalvo JM. AXL - a new player in resistance to HER2 blockade. Cancer Treat Rev 2023;121:102639. 37864955 10.1016/j.ctrv.2023.102639

[46] Rodon J , Postel-Vinay S, Hollebecque A, Nuciforo P, Azaro A, Cattan V, . First-in-human phase I study of oral S49076, a unique MET/AXL/FGFR inhibitor, in advanced solid tumours. Eur J Cancer 2017;81:142–50. 28624695 10.1016/j.ejca.2017.05.007

[47] Felip E , Brunsvig P, Vinolas N, Ponce Aix S, Carcereny Costa E, Dómine Gomez M, . A phase II study of bemcentinib (BGB324), a first-in-class highly selective AXL inhibitor, with pembrolizumab in pts with advanced NSCLC: OS for stage I and preliminary stage II efficacy. J Clin Oncol 2019;37(Suppl 15):9098.

[48] Sooi K , Tan TZ, Kim J-W, Lee JY, Kim B-G, Micklem D, . A phase 1b, multicentre, dose escalation, safety and pharmacokinetics study of tilvestamab (BGB149) in relapsed, platinum-resistant, high-grade serous ovarian cancer (PROC) patients. Br J Cancer 2025;133:896–908. 40696160 10.1038/s41416-025-03090-6 PMC12449448

[49] Shen GG , Briggs E, Crass R, Karumanchi S, Boyle WJ, Baker MG, . Population pharmacokinetic and exposure-response safety analyses of mecbotamab vedotin (BA3011) in patients with advanced solid tumors. J Clin Oncol 2023;41(Suppl 16):e15010.

[50] Pollack S , Conley AP, Tap WD, Yen CC, Charlson J, Davis L, . 53O Results from a phase II part I trial of mecbotamab vedotin (BA3011), a CAB-AXL-ADC, in patients with advanced refractory sarcoma. ESMO Open 2024;9(Suppl 2):102443.

