# Supplementary material for: Phase I/II Study of AXL-Specific Antibody–Drug Conjugate Enapotamab Vedotin in Patients with Advanced Solid Tumors
Source: Cancer Res Commun. 2025 Nov 26;5(11):2066–78. doi: 10.1158/2767-9764.CRC-25-0359 (PMC12648153; doi:10.1158/2767-9764.CRC-25-0359)
Supplement: Table S1 — Key inclusion and exclusion criteria [file crc-25-0359_table_s1_suppst1.docx]

**Supplementary Table S1.** Key inclusion and exclusion criteria.

| **Key Inclusion Criteria** |
| --- |
| 1. For dose-escalation phase: relapsed or refractory cancer of the ovary, cervix, endometrium, thyroid, NSCLC, or melanoma (cutaneous, mucosal, acral or uveal melanoma) who had failed available standard therapy or who were not candidates for standard therapy, and for whom, in the opinion of the investigator, experimental therapy with enapotamab vedotin may have been beneficial.  - For the dose-expansion phase: advanced and/or metastatic cancer with documented disease progression on the last prior therapy, who were not candidates for standard therapy, and for whom, in the opinion of the investigator, experimental therapy with enapotamab vedotin might be beneficial, who had failed the following anticancer therapy as follows: - Dose-expansion cohort 1 (NSCLC patients with classical sensitizing *EGFR* mutations and/or *EGFR* mutations targeted by third-generation TKIs [eg, T790M for osimertinib]):   - NSCLC patients after failure of up to 4 prior treatment regimens containing systemic therapy for metastatic disease; adjuvant and maintenance treatment was considered to be part of 1 treatment regimen.   - Last prior treatment to enrollment should have been an EGFR inhibitor (eg, erlotinib, osimertinib, etc), or a PD-1/PD-L1 inhibitor, or a platinum-based doublet chemotherapy. - Dose-expansion cohorts 2 and 8 (patients with NSCLC without activating *EGFR* mutations or *ALK* rearrangements):   - Patients with NSCLC after failure of no more than 2 lines of therapy, which included a platinum-based chemotherapy and PD-1/PD-L1 inhibitor treatment for advanced (stage IIIA or IIIB) or metastatic disease (stage IV) either in combination or sequentially.   - Last prior treatment to enrollment should have been (either in combination or sequentially) a platinum-based chemotherapy or a PD-1/PD-L1 inhibitor. - Dose-expansion cohort 3 (patients with melanoma with BRAF V600 mutation):   - Patients with cutaneous, acral, or mucosal melanoma after failure of up to 4 prior treatment regimens containing systemic therapy for metastatic disease; adjuvant and maintenance treatment was considered to be part of 1 treatment regimen.   - Last prior treatment to enrollment should have been a BRAF inhibitor (± MEK inhibitor) or a checkpoint inhibitor. - Dose-expansion cohort 4 (patients with melanoma with BRAF V600 wild type):   - Cutaneous, acral, or mucosal melanoma patients after failure of up to 3 prior treatment regimens containing systemic therapy for metastatic disease; adjuvant and maintenance treatment was considered to be part of 1 treatment regimen.   - Last prior treatment to enrollment should have been a checkpoint inhibitor. - Expansion cohort 5 (patients with sarcoma):   - Patients with sarcoma after failure of up to 3 prior treatment regimens containing systemic therapy for metastatic disease. Limited to undifferentiated pleomorphic sarcoma, lipo-, leiomyosarcoma, synovial sarcoma, Ewing’s sarcoma, osteo-, and chondrosarcoma; adjuvant and maintenance treatment was considered part of 1 treatment regimen. - Expansion cohort 6 (patients with metastatic solid tumors, excluding NSCLC, melanoma, ovarian cancer, and sarcoma patients unless having a known *AXL* gene amplification; preferably no more than 8 patients were to be recruited for 1 tumor type):   - Patients with solid tumors (except for NSCLC, melanoma, ovarian cancer, and sarcoma unless having a known *AXL* gene amplification) that had failed a PD-1/PD-L1 inhibitor for metastatic disease.   - Last prior treatment to enrollment should have been an immune checkpoint inhibitor. - Expansion cohort 7 (patients with platinum-resistant ovarian cancer):   - Patients with ovarian cancer with resistance to at least 1 platinum-based therapy defined according to Gynecologic Cancer Intergroup (GCIG). Disease progression during or within 6 months of previous platinum-based chemotherapy included the following 2 categories:     - Primary platinum-resistant: previously untreated patients who had achieved at least a partial response (PR) to platinum-based chemotherapy, but experienced a relapse within a period of >1 and <6 months following treatment completion.     - Secondary platinum-resistant: previously treated patients who had achieved at least a PR with platinum-based therapy as second line treatment, but experienced a relapse within a period of >1 and <6 months following treatment completion.   - Patients with ovarian cancer after failure of at least 2 prior treatment regimens containing systemic therapy but not more than 5 for recurrent disease.     - Limited to invasive epithelial tubo-ovarian carcinoma including malignant serous (restricted to high-grade serous ovarian cancer, carcinosarcoma, and high-grade [or grade ≥3] clear cell/endometrioid/mixed epithelial carcinoma)     - Maintenance treatment (eg, with bevacizumab, poly [ADP-ribose] polymerase inhibitor, PD-1/PD-L1 inhibitor, etc.) was considered part of 1 treatment regimen. Treatments that had to be changed to a similar drug due to toxicity count as 1 regimen (eg, change from carboplatin to cis-platinum [cisplatin] because of allergy, etc).   - Start of screening must have been within 60 days after documented progression. Isolated GCIG CA-125 progression did NOT qualify for trial entry.   - Albumin levels should have been >25 g/L (“NCI-CTCAE grade 2 intermediate”) to allow inclusion.  1. Patient had measurable disease according to RECIST v1.1.  - In all dose-expansion cohorts, the sponsor medical officer’s approval of enrollment was needed if documented progression had not been on measurable disease (ie, symptomatic progression). - In the dose-escalation phase, patients with ovarian cancer could be included based on CA-125 positivity according to the GCIG guideline only if they had a pretreatment sample that was at least twice the upper limit of the reference range and within 2 weeks before starting the treatment. Note: patients were not evaluable by CA-125 if they had received mouse antibodies (unless the assay used has been shown not to be influenced by human anti-mouse antibody) or if there had been medical and/or surgical interference with their peritoneum or pleura during the previous 28 days (eg, paracentesis).  1. In the dose-escalation phase, all patients provided a tumor tissue sample (formalin-fixed paraffin-embedded [FFPE] blocks/slides) from archival tissue or fresh biopsy collected before cycle 1, day 1 (C1D1), preferably derived from advanced disease stage. In the dose-expansion phase, all patients provided a mandatory fresh biopsy (FFPE tissue blocks/slides) at screening (aspirates were not acceptable), which contained tumor tissue and was taken after failure/stop of last prior treatment. In case it was not feasible to meet the required criteria for fresh tumor biopsy, the sponsor medical officer’s approval of enrollment was needed. Furthermore, the latest available archival tumor tissue sample was collected if available. 2. Age ≥18 years. 3. Eastern Cooperative Oncology Group performance status (ECOG PS) of 0 or 1. 4. Life expectancy of at least 3 months |
| **Key Exclusion Criteria** |
| 1. Acute deep vein thrombosis or clinically relevant pulmonary embolism, not stable for at least 4 weeks prior to first enapotamab vedotin administration. 2. History of thromboembolic event(s) and not willing to take thromboembolic prophylaxis. 3. Clinically significant cardiac disease, including:  - Onset of unstable angina within 6 months of signing the ICF. - Acute myocardial infarction within 6 months of the signing the ICF. - Known congestive heart failure (grade III or IV as classified by the New York Heart Association); and/or a known decreased cardiac ejection fraction of <45% and/or baseline QT interval as corrected by Fridericia’s formula (QTcF) >480 msec or uncontrolled atrial fibrillation. - Uncontrolled hypertension defined as systolic blood pressure ≥160 mm Hg and/or diastolic blood pressure ≥100 mm Hg, despite optimal medical management.  1. Ongoing or recent (within 1 year) evidence of significant autoimmune disease that required treatment with systemic immunosuppressive treatments, which might suggest risk for immune-related adverse events (irAEs). 2. History of grade ≥3 irAEs (AEs below grade 3 were to be discussed with the sponsor). 3. History of non-infectious pneumonitis related to prior systemic treatment and that required treatment with steroids within the last 6 months prior to enrollment. If an event of pneumonitis was considered fully resolved more than 6 months prior to trial start (ie, patient had no radiologic evidence of pneumonitis, was asymptomatic, and did not require any steroid treatment), patient could be enrolled. 4. Received granulocyte colony-stimulating factor (G-CSF) or granulocyte/macrophage colony-stimulating factor support 3 weeks prior to first enapotamab vedotin administration. 5. Received a cumulative dose of corticosteroid >150 mg prednisone (or equivalent doses of corticosteroids) within 2 weeks before the first enapotamab vedotin administration. 6. History of grade ≥3 allergic reactions to monoclonal antibody therapy as well as known or suspected allergy or intolerance to any agent given in the course of this trial. 7. Major surgery within 4 weeks before first enapotamab vedotin administration. 8. History of intracerebral arteriovenous malformation, cerebral aneurysm, brain metastases, or stroke. Transient ischemic attack ≥6 months prior to screening was allowed. 9. Any anticancer therapy including small molecules, immunotherapy, chemotherapy monoclonal antibodies, or any other experimental drug within 5 half-lives but at a maximum 4 weeks before first infusion. Exceptions were bisphosphonates, denosumab, and gonadotropin-releasing hormone agonists or antagonists, which could be continued throughout the trial. Toxic effects of prior anticancer therapy considered as chronic, such as chemotherapy-induced fatigue, alopecia, or anorexia of grade ≤2, where further resolution was expected, did not prevent the patient from participation in the trial. 10. Any prior therapy with a conjugated or unconjugated auristatin derivative/vinca-binding site targeting payload (previous treatment with vinca alkaloids was allowed). 11. Radiotherapy within 14 days of first enapotamab vedotin administration. Palliative radiotherapy was allowed. 12. Patients who discontinued treatment due to disease progression within the first 6 weeks of commencing a prior immune checkpoint inhibitor–containing treatment. 13. Known past or current malignancy other than inclusion diagnosis, except for:  - Cervical carcinoma of stage 1B or less. - Non-invasive basal cell or squamous cell skin carcinoma. - Non-invasive, superficial bladder cancer. - Prostate cancer with a current PSA level <0.1 ng/mL. - Breast cancer in BRCA1- or BRCA2-positive ovarian cancer patients. - Any curable cancer with a complete response of >2 years duration.  1. Melanoma patients with a lactate dehydrogenase ≥3× upper limit of normal. 2. Ongoing significant, uncontrolled medical condition including serious, non-healing wound, skin ulcer (of any grade), or bone fracture. 3. Presence of grade ≥2 peripheral neuropathy. 4. Clinically significant active viral, bacterial or fungal infection requiring: intravenous treatment with anti-infective therapy that had been administered less than 2 weeks prior to first dose; or oral treatment with anti-infective therapy that had been administered less than 1 week prior to first dose; prophylactic anti-infective therapy, which was given without clinical symptoms, was allowed (eg, antibiotic prophylaxis prior to dental extraction, etc). 5. Specifically for NSCLC:    - Pulmonary hemorrhage or hemoptysis >2.5 mL blood within 6 weeks unless cause had been addressed and was medically resolved. |
