# Supplementary material for: Phase I/II Study of AXL-Specific Antibody–Drug Conjugate Enapotamab Vedotin in Patients with Advanced Solid Tumors
Source: Cancer Res Commun. 2025 Nov 26;5(11):2066–78. doi: 10.1158/2767-9764.CRC-25-0359 (PMC12648153; doi:10.1158/2767-9764.CRC-25-0359)
Supplement: Table S2 — Representativeness of study participants [file crc-25-0359_table_s2_suppst2.docx]

**Supplementary Table S2.** Representativeness of study participants

| Cancer types/subtypes/stages/condition | Advanced solid tumors^a^ |
| --- | --- |
| Considerations related to: |  |
| Sex | In 2025, it is estimated that 10.9 million new cases of cancer (all types) will occur in male patients and 10.4 million new cases will occur in female patients globally.^1^ Most of these cancers are expected to be solid tumor types, with a slightly higher incidence in men but overall representing approximately a 50:50 distribution^1^ |
| Age | Worldwide in 2020, older adults accounted for 64% of new cancer cases.^2^ In the United States in 2022, approximately 2.1 million patients were diagnosed with cancer; of these patients, 665,573 (31.7%) were aged 65–74 years and 575,875 (27.4%) were aged >75 years.^3^ The median age of cancer diagnosis reported in the United States across all tumor types is 67 years^4^ |
| Race/ethnicity | In 2022, the rate of new cancers in the United States per 100,000 people was 458.6 for White non-Hispanic populations (1,355,428 cases), 445.4 for Black non-Hispanic populations (204,615 cases), 398.3 for American Indian and Alaska native non-Hispanic populations (11,740 cases), 352.6 for Hispanic populations (177,254 cases), and 300.7 for Asian and Pacific Islander non-Hispanic populations (72,114 cases)^5^ |
| Geography | Our study includes patients from Belgium, Denmark, Netherlands, Spain, United Kingdom, and United States (NCT02988817) |
| Overall representativeness of this study | Patients treated with enapotamab vedotin in our study were generally representative of the wider distribution of patients with advanced solid tumors. In our study, 28%–59% of patients were female across the dose-expansion cohorts (excluding ovarian cancer). Patients aged >65 years accounted for 12.5%–56.4%, with the median age of patients ranging from 58.0–66.0 years (overall range: 23.0-81.0). Overall, our study population was predominantly White (75%–96% across the dose-expansion cohorts). A relatively wide range of advanced solid tumors was included in our study with a diverse population. Differences in the patient populations can be attributed to a relatively small sample size in this phase 1/2 study |

^a^Eligible tumor types: relapsed or refractory NSCLC, melanoma, sarcoma, ovarian cancer, cervical cancer, endometrial cancer, thyroid cancer, and other solid tumors.

Abbreviation: NSCLC, non-small cell lung cancer.

**References**

1. World Health Organization. International Agency for Research on Cancer. Cancer tomorrow. Available at: https://gco.iarc.fr/tomorrow/en/dataviz/isotype?types=0&single_unit=500000&populations=900&group_populations=0&multiple_populations=0&years=2025&sexes=1. Accessed September 23, 2025.
2. Li L, Shan T, Zhang D, Ma F. Nowcasting and forecasting global aging and cancer burden: analysis of data from the GLOBOCAN and Global Burden of Disease Study. *J Natl Cancer Cent.* **2024**;4:223-232.
3. US Centers for Disease Control and Prevention. Highlights from 2025 U.S. Cancer Statistics. Available at: https://www.cdc.gov/united-states-cancer-statistics/publications/uscs-highlights.html. Accessed October 5, 2025.
4. National Cancer Institute. Age and cancer risk. Available at: https://www.cancer.gov/about-cancer/causes-prevention/risk/age#:~:text=According%20to%20the%20most%20recent,diagnosed%20in%20this%20age%20group. Accessed September 24, 2025.
5. US Centers for Disease Control and Prevention. US cancer statistics: data visualizations. June 2025. Available at: https://www.cdc.gov/cancer/dataviz. Accessed September 25, 2025.
