# Supplementary material for: Phase I/II Study of AXL-Specific Antibody–Drug Conjugate Enapotamab Vedotin in Patients with Advanced Solid Tumors
Source: Cancer Res Commun. 2025 Nov 26;5(11):2066–78. doi: 10.1158/2767-9764.CRC-25-0359 (PMC12648153; doi:10.1158/2767-9764.CRC-25-0359)
Supplement: Table S3 — Safety summary: TEAEs in ≥5% of patients overall and grade ≥3 TEAEs in dose-escalation phase (Q3W schedule) [file crc-25-0359_table_s3_suppst3.docx]

**Supplementary Table S3.** Safety summary: TEAEs in ≥5% of patients overall and grade ≥3 TEAEs in dose-escalation phase (Q3W schedule).

| **n (%)** | **0.3 mg/kg (n=1)** | **0.6 mg/kg (n=1)** | **1.0 mg/kg (n=3)** | **1.5 mg/kg (n=3)** | **2.0 mg/kg (n=10)** | **2.2 mg/kg (n=11)** | **2.4 mg/kg (n=3)** | **Total (N=32)** |
| --- | --- | --- | --- | --- | --- | --- | --- | --- |
| ≥1 TEAE | 1 (100.0) | 1 (100.0) | 3 (100.0) | 3 (100.0) | 10 (100.0) | 11 (100.0) | 3 (100.0) | 32 (100.0) |
| Related TEAE | 1 (100.0) | 1 (100.0) | 3 (100.0) | 3 (100.0) | 10 (100.0) | 10 (90.9) | 3 (100.0) | 31 (96.9) |
| Infusion-related TEAE | 0 | 0 | 0 | 1 (33.3) | 0 | 0 | 0 | 1 (3.1) |
| TEAE leading to discontinuation | 0 | 0 | 0 | 0 | 3 (30.0) | 2 (18.2) | 3 (100.0) | 8 (25) |
| TEAE leading to treatment interruption | 0 | 0 | 0 | 1 (33.3) | 3 (30.0) | 1 (9.1) | 0 | 5 (15.6) |
| TEAE leading to dose reduction | 0 | 0 | 0 | 0 | 3 (30.0) | 2 (18.2) | 2 (66.7) | 7 (21.9) |
| Grade 3/4 TEAE | 0 | 0 | 0 | 2 (66.7) | 7 (70.0) | 9 (81.8) | 3 (100.0) | 21 (65.6) |
| Related grade 3/4 TEAE | 0 | 0 | 0 | 1 (33.3) | 7 (70.0) | 5 (45.5) | 3 (100.0) | 16 (50.0) |
| **TEAEs in ≥5% of Patients Overall** | **0.3 mg/kg (n=1)** | **0.6 mg/kg (n=1)** | **1.0 mg/kg (n=3)** | **1.5 mg/kg (n=3)** | **2.0 mg/kg (n=10)** | **2.2 mg/kg (n=11)** | **2.4 mg/kg (n=3)** | **Total (N=32)** |
| Fatigue | 1 (100.0) | 1 (100.0) | 2 (66.7) | 3 (100.0) | 5 (50.0) | 8 (72.7) | 3 (100.0) | 23 (71.9) |
| Nausea | 1 (100.0) | 1 (100.0) | 1 (33.3) | 2 (66.7) | 6 (60.0) | 7 (63.6) | 3 (100.0) | 21 (65.6) |
| Constipation | 0 | 0 | 1 (33.3) | 1 (33.3) | 9 (90.0) | 7 (63.6) | 2 (66.7) | 20 (62.5) |
| Vomiting | 0 | 1 (100.0) | 0 | 2 (66.7) | 6 (60.0) | 7 (63.6) | 3 (100.0) | 19 (59.4) |
| Decreased appetite | 0 | 1 (100.0) | 0 | 2 (66.7) | 3 (30.0) | 8 (72.7) | 2 (66.7) | 16 (50.0) |
| Diarrhea | 0 | 0 | 0 | 1 (33.3) | 5 (50.0) | 7 (63.6) | 1 (33.3) | 14 (43.8) |
| Abdominal pain | 0 | 0 | 0 | 0 | 6 (60.0) | 4 (36.4) | 2 (66.7) | 12 (37.5) |
| Neutropenia | 0 | 0 | 1 (33.3) | 1 (33.3) | 4 (40.0) | 2 (18.2) | 2 (66.7) | 10 (31.3) |
| Alopecia | 0 | 0 | 0 | 1 (33.3) | 2 (20.0) | 3 (27.3) | 3 (100.0) | 9 (28.1) |
| Anemia | 0 | 0 | 1 (33.3) | 1 (33.3) | 4 (40.0) | 2 (18.2) | 1 (33.3) | 9 (28.1) |
| Hypokalemia | 0 | 0 | 0 | 2 (66.7) | 3 (30.0) | 3 (27.3) | 1 (33.3) | 9 (28.1) |
| Peripheral sensory neuropathy | 0 | 1 (100.0) | 0 | 0 | 5 (50.0) | 2 (18.2) | 1 (33.3) | 9 (28.1) |
| Aspartate aminotransferase increased | 0 | 1 (100.0) | 0 | 0 | 3 (30.0) | 3 (27.3) | 1 (33.3) | 8 (25.0) |
| Back pain | 0 | 0 | 2 (66.7) | 1 (33.3) | 2 (20.0) | 3 (27.3) | 0 | 8 (25.0) |
| Alanine aminotransferase increased | 0 | 1 (100.0) | 0 | 0 | 1 (10.0) | 4 (36.4) | 1 (33.3) | 7 (21.9) |
| Abdominal pain upper | 0 | 0 | 0 | 1 (33.3) | 1 (10.0) | 2 (18.2) | 2 (66.7) | 6 (18.8) |
| Cough | 0 | 1 (100.0) | 1 (33.3) | 0 | 1 (10.0) | 1 (9.1) | 2 (66.7) | 6 (18.8) |
| Dyspepsia | 0 | 0 | 0 | 1 (33.3) | 2 (20.0) | 2 (18.2) | 1 (33.3) | 6 (18.8) |
| Dyspnea | 0 | 0 | 0 | 1 (33.3) | 0 | 3 (27.3) | 2 (66.7) | 6 (18.8) |
| Gamma glutamyl transferase increased | 0 | 0 | 0 | 0 | 2 (20.0) | 3 (27.3) | 1 (33.3) | 6 (18.8) |
| Headache | 1 (100.0) | 0 | 0 | 0 | 1 (10.0) | 4 (36.4) | 0 | 6 (18.8) |
| Weight decreased | 0 | 0 | 0 | 0 | 2 (20.0) | 4 (36.4) | 0 | 6 (18.8) |
| Hypomagnesemia | 0 | 0 | 0 | 1 (33.3) | 2 (20.0) | 2 (18.2) | 0 | 5 (15.6) |
| Hyponatremia | 0 | 1 (100.0) | 0 | 0 | 0 | 3 (27.3) | 1 (33.3) | 5 (15.6) |
| Pyrexia | 0 | 0 | 1 (33.3) | 1 (33.3) | 2 (20.0) | 0 | 1 (33.3) | 5 (15.6) |
| Blood alkaline phosphatase increased | 0 | 1 (100.0) | 0 | 0 | 1 (10.0) | 2 (18.2) | 0 | 4 (12.5) |
| Dizziness | 0 | 0 | 1 (33.3) | 0 | 1 (10.0) | 1 (9.1) | 1 (33.3) | 4 (12.5) |
| Dry eye | 1 (100.0) | 0 | 0 | 1 (33.3) | 0 | 1 (9.1) | 1 (33.3) | 4 (12.5) |
| Insomnia | 0 | 0 | 0 | 1 (33.3) | 0 | 2 (18.2) | 0 | 4 (12.5) |
| Myalgia | 1 (100.0) | 0 | 1 (33.3) | 2 (66.7) | 0 | 0 | 0 | 4 (12.5) |
| Urinary tract infection | 0 | 0 | 0 | 1 (33.3) | 0 | 2 (18.2) | 1 (33.3) | 4 (12.5) |
| Arthralgia | 1 (100.0) | 0 | 0 | 0 | 0 | 1 (9.1) | 1 (33.3) | 3 (9.4) |
| Cystitis | 0 | 0 | 1 (33.3) | 1 (33.3) | 0 | 1 (9.1) | 0 | 3 (9.4) |
| Dry skin | 0 | 0 | 0 | 1 (33.3) | 0 | 1 (9.1) | 1 (33.3) | 3 (9.4) |
| Dysgeusia | 0 | 0 | 0 | 0 | 0 | 3 (27.3) | 0 | 3 (9.4) |
| Hyperkalemia | 0 | 0 | 0 | 1 (33.3) | 0 | 2 (18.2) | 0 | 3 (9.4) |
| Hypertension | 0 | 0 | 0 | 1 (33.3) | 1 (10.0) | 1 (9.1) | 0 | 3 (9.4) |
| Intestinal obstruction | 0 | 0 | 0 | 1 (33.3) | 1 (10.0) | 1 (9.1) |  | 3 (9.4) |
| Leukopenia | 0 | 0 | 0 | 0 | 2 (20.0) | 0 | 1 (33.3) | 3 (9.4) |
| Lipase increased | 1 (100.0) | 0 | 0 | 0 | 1 (10.0) | 0 | 1 (33.3) | 3 (9.4) |
| Nasopharyngitis | 0 | 0 | 0 | 1 (33.3) | 1 (10.0) | 0 | 1 (33.3) | 3 (9.4) |
| Peripheral sensorimotor neuropathy | 0 | 0 | 0 | 0 | 1 (10.0) | 0 | 2 (66.7) | 3 (9.4) |
| Pruritus | 0 | 0 | 0 | 1 (33.3) | 1 (10.0) | 1 (9.1) | 0 | 3 (9.4) |
| Rash | 0 | 0 | 1 (33.3) | 0 | 1 (10.0) | 1 (9.1) | 0 | 3 (9.4) |
| Sinus tachycardia | 0 | 0 | 0 | 0 | 2 (20.0) | 0 | 1 (33.3) | 3 (9.4) |
| Upper respiratory tract  infection | 0 | 0 | 1 (33.3) | 0 | 1 (10.0) | 1 (9.1) | 0 | 3 (9.4) |
| Abdominal distension | 0 | 0 | 0 | 1 (33.3) | 0 | 0 | 1 (33.3) | 2 (6.3) |
| Amylase increased | 0 | 0 | 0 |  | 2 (20.0) | 0 | 0 | 2 (6.3) |
| Anxiety | 0 | 0 | 0 | 1 (33.3) | 1 (10.0) | 0 | 0 | 2 (6.3) |
| Asthenia | 0 | 0 | 0 | 1 (33.3) | 0 | 1 (9.1) | 0 | 2 (6.3) |
| Blood creatinine increased | 0 | 1 (100.0) | 0 | 0 | 1 (10.0) | 0 | 0 | 2 (6.3) |
| Dyspnea exertional | 0 | 1 (100.0) | 0 | 0 | 1 (10.0) | 0 | 0 | 2 (6.3) |
| Gastroenteritis viral | 0 | 0 | 0 | 0 | 1 (10.0) | 0 | 1 (33.3) | 2 (6.3) |
| Hot flush | 0 | 0 | 0 | 1 (33.3) | 0 | 1 (9.1) | 0 | 2 (6.3) |
| Lymphopenia | 0 | 1 (100.0) | 0 | 0 | 1 (10.0) | 0 | 0 | 2 (6.3) |
| Muscle spasms | 0 | 1 (100.0) | 0 | 0 | 0 | 0 | 1 (33.3) | 2 (6.3) |
| Neck pain | 0 | 0 | 1 (33.3) | 0 | 0 | 1 (9.1) | 0 | 2 (6.3) |
| Neuralgia | 0 | 0 | 1 (33.3) | 0 | 0 | 0 | 1 (33.3) | 2 (6.3) |
| Edema peripheral | 0 | 0 | 0 | 0 | 0 | 1 (9.1) | 1 (33.3) | 2 (6.3) |
| Oropharyngeal pain | 0 | 0 | 0 | 0 | 0 | 1 (9.1) | 1 (33.3) | 2 (6.3) |
| Restless legs syndrome | 0 | 0 | 0 | 0 | 0 | 1 (9.1) | 1 (33.3) | 2 (6.3) |
| Thrombocytopenia | 0 | 0 | 1 (33.3) | 0 | 1 (10.0) | 0 | 0 | 2 (6.3) |
| Vaginal hemorrhage | 0 | 0 | 1 (33.3) | 1 (33.3) | 0 | 0 | 0 | 2 (6.3) |
| Vertigo | 0 | 0 | 0 | 1 (33.3) | 0 | 1 (9.1) | 0 | 2 (6.3) |
| **Grade ≥3 TEAEs** | **0.3 mg/kg (n=1)** | **0.6 mg/kg (n=1)** | **1.0 mg/kg (n=3)** | **1.5 mg/kg (n=3)** | **2.0 mg/kg (n=10)** | **2.2 mg/kg (n=11)** | **2.4 mg/kg (n=3)** | **Total (N=32)** |
| ≥1 Grade ≥3 TEAE | 0 | 0 | 0 | 2 (66.7) | 7 (70.0) | 9 (81.8) | 3 (100.0) | 21 (65.6) |
| Neutropenia | 0 | 0 | 0 | 0 | 4 (40.0) | 1 (9.1) | 2 (66.7) | 7 (21.9) |
| Constipation | 0 | 0 | 0 | 0 | 3 (30.0) | 1 (9.1) | 1 (33.3) | 5 (15.6) |
| Gamma-glutamyl transferase increased | 0 | 0 | 0 | 0 | 0 | 2 (18.2) | 1 (33.3) | 3 (9.4) |
| Diarrhea | 0 | 0 | 0 | 0 | 0 | 2 (18.2) | 0 | 2 (6.3) |
| Hypokalemia | 0 | 0 | 0 | 0 | 0 | 1 (9.1) | 1 (33.3) | 2 (6.3) |
| Malignant neoplasm progression | 0 | 0 | 0 | 0 | 0 | 2 (18.2) | 0 | 2 (6.3) |
| Vomiting | 0 | 0 | 0 | 0 | 1 (10.0) | 1 (9.1) | 0 | 2 (6.3) |
| Abdominal pain upper | 0 | 0 | 0 | 0 | 0 | 1 (9.1) | 0 | 1 (3.1) |
| Activated partial thromboplastin time prolonged | 0 | 0 | 0 | 0 | 0 | 0 | 1 (33.3) | 1 (3.1) |
| Alanine aminotransferase increased | 0 | 0 | 0 | 0 | 0 | 1 (9.1) | 0 | 1 (3.1) |
| Amylase increased | 0 | 0 | 0 | 0 | 1 (10.0) | 0 | 0 | 1 (3.1) |
| Anemia | 0 | 0 | 0 | 0 | 0 | 1 (9.1) | 0 | 1 (3.1) |
| Arthralgia | 0 | 0 | 0 | 0 | 0 | 1 (9.1) | 0 | 1 (3.1) |
| Aspartate aminotransferase increased | 0 | 0 | 0 | 0 | 0 | 1 (9.1) | 0 | 1 (3.1) |
| Bacteremia | 0 | 0 | 0 | 1 (33.3) | 0 | 0 | 0 | 1 (3.1) |
| Dehydration | 0 | 0 | 0 | 0 | 1 (10.0) | 0 | 0 | 1 (3.1) |
| Fatigue | 0 | 0 | 0 | 0 | 0 | 1 (9.1) | 0 | 1 (3.1) |
| Hypertriglyceridemia | 0 | 0 | 0 | 0 | 0 | 1 (9.1) | 0 | 1 (3.1) |
| Hypomagnesemia | 0 | 0 | 0 | 0 | 1 (10.0) | 0 | 0 | 1 (3.1) |
| Hyponatremia | 0 | 0 | 0 | 0 | 0 | 1 (9.1) | 0 | 1 (3.1) |
| Ileus | 0 | 0 | 0 | 1 (33.3) | 0 | 0 | 0 | 1 (3.1) |
| Intestinal obstruction | 0 | 0 | 0 | 1 (33.3) | 0 | 0 | 0 | 1 (3.1) |
| Leukopenia | 0 | 0 | 0 | 0 | 1 (10.0) | 0 | 0 | 1 (3.1) |
| Lipase increased | 0 | 0 | 0 | 0 | 0 | 0 | 1 (33.3) | 1 (3.1) |
| Peripheral sensorimotor neuropathy | 0 | 0 | 0 | 0 | 0 | 0 | 1 (33.3) | 1 (3.1) |
| Peripheral sensory neuropathy | 0 | 0 | 0 | 0 | 0 | 0 | 1 (33.3) | 1 (3.1) |

Abbreviations: Q3W, once every 3 weeks; TEAE, treatment-emergent adverse event.
