# Supplementary material for: Phase I/II Study of AXL-Specific Antibody–Drug Conjugate Enapotamab Vedotin in Patients with Advanced Solid Tumors
Source: Cancer Res Commun. 2025 Nov 26;5(11):2066–78. doi: 10.1158/2767-9764.CRC-25-0359 (PMC12648153; doi:10.1158/2767-9764.CRC-25-0359)
Supplement: Table S4 — Safety summary: TEAEs in ≥5% of patients overall in dose-escalation phase (3Q4W schedule) [file crc-25-0359_table_s4_suppst4.docx]

**Supplementary Table S4.** Safety summary: TEAEs in ≥5% of patients overall in dose-escalation phase (3Q4W schedule).

| **n (%)** | **0.6 mg/kg (n=3)** | **0.8 mg/kg (n=3)** | **1.0 mg/kg (n=6)** | **1.2 mg/kg (n=3)** | **Total (N=15)** |
| --- | --- | --- | --- | --- | --- |
| ≥1 TEAE | 3 (100.0) | 3 (100.0) | 6 (100.0) | 3 (100.0) | 15 (100.0) |
| Related TEAE | 3 (100.0) | 3 (100.0) | 6 (100.0) | 3 (100.0) | 15 (100.0) |
| Infusion-related TEAE | 0 | 2 (66.7) | 1 (16.7) | 0 | 3 (20.0) |
| TEAE leading to discontinuation | 0 | 0 | 1 (16.7) | 2 (66.7) | 3 (20.0) |
| TEAE leading to treatment interruption | 0 | 1 (33.3) | 1 (16.7) | 1 (33.3) | 3 (20.0) |
| TEAE leading to dose reduction | 0 | 0 | 1 (16.7) | 0 | 1 (6.7) |
| Grade 3/4 TEAE | 1 (33.3) | 0 | 3 (50.0) | 3 (100.0) | 7 (46.7) |
| Related grade 3/4 TEAE | 0 | 0 | 1 (16.7) | 2 (66.7) | 3 (20.0) |
| **TEAEs in ≥5% of Patients Overall** | **0.6 mg/kg (n=3)** | **0.8 mg/kg (n=3)** | **1.0 mg/kg (n=6)** | **1.2 mg/kg (n=3)** | **Total (N=15)** |
| Fatigue | 1 (33.3) | 1 (33.3) | 5 (83.3) | 3 (100.0) | 10 (66.7) |
| Constipation | 2 (66.7) | 2 (66.7) | 4 (66.7) | 0 | 8 (53.3) |
| Diarrhea | 2 (66.7) | 0 | 4 (66.7) | 2 (66.7) | 8 (53.3) |
| Nausea | 0 | 2 (66.7) | 4 (66.7) | 2 (66.7) | 8 (53.3) |
| Anemia | 1 (33.3) | 0 | 3 (50.0) | 1 (33.3) | 5 (33.3) |
| Decreased appetite | 1 (33.3) | 0 | 3 (50.0) | 1 (33.3) | 5 (33.3) |
| Abdominal pain | 0 | 0 | 2 (33.3) | 1 (33.3) | 3 (20.0) |
| Aspartate aminotransferase increased | 0 | 1 (33.3) | 1 (16.7) | 1 (33.3) | 3 (20.0) |
| Peripheral sensory neuropathy | 1 (33.3) | 0 | 2 (33.3) | 0 | 3 (20.0) |
| Pyrexia | 1 (33.3) | 0 | 2 (33.3) | 0 | 3 (20.0) |
| Vomiting | 1 (33.3) | 0 | 1 (16.7) | 1 (33.3) | 3 (20.0) |
| Alanine aminotransferase increased | 0 | 1 (33.3) | 0 | 1 (33.3) | 2 (13.3) |
| Alopecia | 0 | 0 | 2 (33.3) | 0 | 2 (13.3) |
| Arthralgia | 0 | 0 | 2 (33.3) | 0 | 2 (13.3) |
| Gamma glutamyl transferase increased | 0 | 0 | 1 (16.7) | 1 (33.3) | 2 (13.3) |
| Hyperglycemia | 0 | 0 | 0 | 2 (66.7) | 2 (13.3) |
| Hypertension | 0 | 1 (33.3) | 1 (16.7) | 0 | 2 (13.3) |
| Hypoalbuminemia | 0 | 0 | 1 (16.7) | 1 (33.3) | 2 (13.3) |
| Hypokalemia | 0 | 0 | 1 (16.7) | 1 (33.3) | 2 (13.3) |
| Hypomagnesemia | 0 | 0 | 1 (16.7) | 1 (33.3) | 2 (13.3) |
| Myalgia | 0 | 0 | 2 (33.3) | 0 | 2 (13.3) |
| Neutropenia | 0 | 0 | 1 (16.7) | 1 (33.3) | 2 (13.3) |
| Oral candidiasis | 0 | 0 | 1 (16.7) | 1 (33.3) | 2 (13.3) |
| Urinary tract infection | 0 | 0 | 2 (33.3) | 0 | 2 (13.3) |
| Weight decreased | 0 | 0 | 2 (33.3) | 0 | 2 (13.3) |
| Abdominal discomfort | 0 | 0 | 1 (16.7) | 0 | 1 (6.7) |
| Abdominal pain upper | 0 | 0 | 1 (16.7) | 0 | 1 (6.7) |
| Asthenia | 0 | 1 (33.3) | 0 | 0 | 1 (6.7) |
| Back pain | 0 | 0 | 1 (16.7) | 0 | 1 (6.7) |
| Blood alkaline phosphatase increased | 0 | 0 | 0 | 1 (33.3) | 1 (6.7) |
| Blood bilirubin increased | 0 | 0 | 0 | 1 (33.3) | 1 (6.7) |
| Blood creatinine increased | 0 | 0 | 1 (16.7) | 0 | 1 (6.7) |
| Blood magnesium decreased | 0 | 0 | 1 (16.7) | 0 | 1 (6.7) |
| Blood sodium decreased | 1 (33.3) | 0 | 0 | 0 | 1 (6.7) |
| Blood triglycerides increased | 0 | 0 | 1 (16.7) | 0 | 1 (6.7) |
| Bone pain | 0 | 1 (33.3) | 0 | 0 | 1 (6.7) |
| Borderline glaucoma | 0 | 1 (33.3) | 0 | 0 | 1 (6.7) |
| Chills | 0 | 0 | 1 (16.7) | 0 | 1 (6.7) |
| Cough | 0 | 1 (33.3) | 0 | 0 | 1 (6.7) |
| Cystitis | 0 | 0 | 1 (16.7) | 0 | 1 (6.7) |
| Deep vein thrombosis | 0 | 0 | 0 | 1 (33.3) | 1 (6.7) |
| Dyspepsia | 0 | 0 | 1 (16.7) | 0 | 1 (6.7) |
| Dysphagia | 0 | 0 | 1 (16.7) | 0 | 1 (6.7) |
| Febrile neutropenia | 0 | 0 | 0 | 1 (33.3) | 1 (6.7) |
| Flank pain | 0 | 0 | 1 (16.7) | 0 | 1 (6.7) |
| Gastroesophageal reflux disease | 0 | 0 | 0 | 1 (33.3) | 1 (6.7) |
| Hepatic steatosis | 0 | 0 | 0 | 1 (33.3) | 1 (6.7) |
| Hernia pain | 1 (33.3) | 0 | 0 | 0 | 1 (6.7) |
| Hyperbilirubinemia | 0 | 0 | 0 | 1 (33.3) | 1 (6.7) |
| Hypercalcemia | 0 | 1 (33.3) | 0 | 0 | 1 (6.7) |
| Hyperuricemia | 0 | 0 | 0 | 1 (33.3) | 1 (6.7) |
| Hypocalcemia | 0 | 0 | 0 | 1 (33.3) | 1 (6.7) |
| Hyponatremia | 0 | 0 | 0 | 1 (33.3) | 1 (6.7) |
| Hypophosphatemia | 0 | 0 | 0 | 1 (33.3) | 1 (6.7) |
| Infection | 0 | 1 (33.3) | 0 | 0 | 1 (6.7) |
| Leukocytosis | 0 | 0 | 0 | 1 (33.3) | 1 (6.7) |
| Lipase increased | 0 | 0 | 1 (16.7) | 0 | 1 (6.7) |
| Medical device discomfort | 1 (33.3) | 0 | 0 | 0 | 1 (6.7) |
| Nocturia | 0 | 0 | 1 (16.7) | 0 | 1 (6.7) |
| Nodule | 1 (33.3) | 0 | 0 | 0 | 1 (6.7) |
| Peripheral edema | 0 | 0 | 1 (16.7) | 0 | 1 (6.7) |
| Pain in extremity | 0 | 0 | 1 (16.7) | 0 | 1 (6.7) |
| Paraesthesia | 0 | 0 | 1 (16.7) | 0 | 1 (6.7) |
| Presyncope | 0 | 0 | 1 (16.7) | 0 | 1 (6.7) |
| Pulmonary embolism | 0 | 0 | 0 | 1 (33.3) | 1 (6.7) |
| Rash | 1 (33.3) | 0 | 0 | 0 | 1 (6.7) |
| Restless legs syndrome | 0 | 0 | 1 (16.7) | 0 | 1 (6.7) |
| Sepsis | 0 | 0 | 0 | 1 (33.3) | 1 (6.7) |
| Tachycardia | 0 | 0 | 1 (16.7) | 0 | 1 (6.7) |
| Thrombocytopenia | 0 | 0 | 0 | 1 (33.3) | 1 (6.7) |
| Vaginal hemorrhage | 0 | 1 (33.3) | 0 | 0 | 1 (6.7) |
| Vertigo | 0 | 0 | 1 (16.7) | 0 | 1 (6.7) |
| Vision blurred | 0 | 0 | 1 (16.7) | 0 | 1 (6.7) |
| Visual impairment | 0 | 0 | 1 (16.7) | 0 | 1 (6.7) |
| White blood cell count decreased | 0 | 0 | 1 (16.7) | 0 | 1 (6.7) |
| **Grade ≥3 TEAEs** | **0.6 mg/kg (n=3)** | **0.8 mg/kg (n=3)** | **1.0 mg/kg (n=6)** | **1.2 mg/kg (n=3)** | **Total (N=15)** |
| ≥1 Grade ≥3 TEAE | 1 (33.3) | 0 | 3 (50.0) | 3 (100.0) | 7 (46.7) |
| Diarrhea | 1 (33.3) | 0 | 2 (33.3) | 1 (33.3) | 4 (26.7) |
| Anemia | 0 | 0 | 2 (33.3) | 1 (33.3) | 3 (20.0) |
| Fatigue | 0 | 0 | 1 (16.7) | 1 (33.3) | 2 (13.3) |
| Constipation | 0 | 0 | 1 (16.7) | 0 | 1 (6.7) |
| Deep vein thrombosis | 0 | 0 | 0 | 1 (33.3) | 1 (6.7) |
| Febrile neutropenia | 0 | 0 | 0 | 1 (33.3) | 1 (6.7) |
| Flank pain | 0 | 0 | 1 (16.7) | 0 | 1 (6.7) |
| Gamma glutamyl transferase increased | 0 | 0 | 0 | 1 (33.3) | 1 (6.7) |
| Hyperbilirubinemia | 0 | 0 | 0 | 1 (33.3) | 1 (6.7) |
| Hyperglycemia | 0 | 0 | 0 | 1 (33.3) | 1 (6.7) |
| Hypophosphatemia | 0 | 0 | 0 | 1 (33.3) | 1 (6.7) |
| Pulmonary embolism | 0 | 0 | 0 | 1 (33.3) | 1 (6.7) |
| Sepsis | 0 | 0 | 0 | 1 (33.3) | 1 (6.7) |

Abbreviations: 3Q4W, 3 weekly doses every 4 weeks; TEAE, treatment-emergent adverse event.
