# Supplementary material for: Phase I/II Study of AXL-Specific Antibody–Drug Conjugate Enapotamab Vedotin in Patients with Advanced Solid Tumors
Source: Cancer Res Commun. 2025 Nov 26;5(11):2066–78. doi: 10.1158/2767-9764.CRC-25-0359 (PMC12648153; doi:10.1158/2767-9764.CRC-25-0359)
Supplement: Table S5 — Safety summary: TEAEs in ≥20% of patients in any cohort in dose-expansion phase [file crc-25-0359_table_s5_suppst5.docx]

**Supplementary Table S5.** Safety summary: TEAEs in ≥20% of patients in any cohort in dose-expansion phase.

|  | **NSCLC** | | | | **Melanoma** | | **Sarcoma** | **Solid Tumor** | **Ovarian** |
| --- | --- | --- | --- | --- | --- | --- | --- | --- | --- |
| **Cohort** | **1** | **2** | **2** | **8** | **3** | **4** | **5** | **6** | **7** |
| **Dose** | **Q3W 2.2 mg/kg (n=22)** | **Q3W 2.2 mg/kg (n=56)** | **Q3W 1.8 mg/kg (n=20)** | **3Q4W  1.0 mg/kg (n=26)** | **Q3W  2.2 mg/kg (n=16)** | **Q3W 2.2 mg/kg (n=25)** | **Q3W 2.2 mg/kg (n=25)** | **3Q4W 1.0 mg/kg (n=44)** | **Q3W 2.2 mg/kg (n=25)** |
| ≥1 TEAE | 22 (100.0) | 56 (100.0) | 20 (100.0) | 26 (100.0) | 16 (100.0) | 25 (100.0) | 25 (100.0) | 44 (100.0) | 25 (100.0) |
| Related TEAE | 21 (95.5) | 52 (92.9) | 16 (80.0) | 19 (73.1) | 15 (93.8) | 24 (96.0) | 24 (96.0) | 38 (86.4) | 23 (92.0) |
| Infusion-related TEAE | 0 | 2 (3.6) | 1 (5.0) | 0 | 0 | 1 (4.0) | 2 (8.0) | 2 (4.5) | 0 |
| TEAE leading to discontinuation | 4 (18.2) | 19 (33.9) | 3 (15.0) | 3 (11.5) | 2 (12.5) | 9 (36.0) | 7 (28.0) | 11 (25.0) | 5 (20.0) |
| TEAE leading to treatment interruption | 8 (36.4) | 19 (33.9) | 4 (20.0) | 10 (38.5) | 3 (18.8) | 6 (24.0) | 5 (20.0) | 15 (34.1) | 9 (36.0) |
| TEAE leading to dose reduction | 7 (31.8) | 11 (19.6) | 7 (35.0) | 8 (30.8) | 7 (43.8) | 7 (28.0) | 6 (24.0) | 12 (27.3) | 4 (16.0) |
| Grade 3/4 TEAE | 15 (68.2) | 40 (71.4) | 14 (70.0) | 13 (50.0) | 6 (37.5) | 18 (72.0) | 16 (64.0) | 21 (47.7) | 15 (60.0) |
| Related grade 3/4 TEAE | 9 (40.9) | 28 (50.0) | 9 (45.0) | 7 (26.9) | 6 (37.5) | 13 (52.0) | 12 (48.0) | 12 (27.3) | 10 (40.0) |
| Related grade 3/4 TEAE | 4 (18.2) | 9 (16.1) | 3 (15.0) | 0 | 0 | 3 (12.0) | 4 (16.0) | 5 (11.4) | 3 (12.0) |
| TEAEs in ≥20% of patients (any cohort) | | | | | | | | | |
| Fatigue | 12 (54.5) | 33 (58.9) | 11 (55.0) | 14 (53.8) | 8 (50.0) | 17 (68.0) | 14 (56.0) | 23 (52.3) | 13 (52.0) |
| Nausea | 14 (63.6) | 26 (46.4) | 6 (30.0) | 11 (42.3) | 10 (62.5) | 11 (44.0) | 15 (60.0) | 13 (29.5) | 15 (60.0) |
| Constipation | 12 (54.5) | 34 (60.7) | 9 (45.0) | 11 (42.3) | 9 (56.3) | 15 (60.0) | 14 (56.0) | 12 (27.3) | 7 (28.0) |
| Diarrhea | 5 (22.7) | 21 (37.5) | 6 (30.0) | 11 (42.3) | 8 (50.0) | 10 (40.0) | 10 (40.0) | 13 (29.5) | 9 (36.0) |
| Decreased appetite | 8 (36.4) | 26 (46.4) | 7 (35.0) | 12 (46.2) | 8 (50.0) | 5 (20.0) | 8 (32.0) | 14 (31.8) | 7 (28.0) |
| Abdominal pain | 4 (18.2) | 11 (19.6) | 3 (15.0) | 7 (26.9) | 4 (25.0) | 8 (32.0) | 5 (20.0) | 10 (22.7) | 9 (36.0) |
| Peripheral sensory neuropathy | 9 (40.9) | 11 (19.6) | 3 (15.0) | 6 (23.1) | 2 (12.5) | 4 (16.0) | 7 (28.0) | 11 (25.0) | 6 (24.0) |
| Vomiting | 7 (31.8) | 18 (32.1) | 4 (20.0) | 7 (26.9) | 3 (18.8) | 4 (16.0) | 6 (24.0) | 9 (20.5) | 8 (32.0) |
| Alopecia | 4 (18.2) | 14 (25.0) | 2 (10.0) | 6 (23.1) | 5 (31.3) | 7 (28.0) | 8 (32.0) | 3 (6.8) | 9 (36.0) |
| Neutropenia | 3 (13.6) | 8 (14.3) | 3 (15.0) | 0 | 2 (12.5) | 7 (28.0) | 7 (28.0) | 2 (4.5) | 9 (36.0) |
| ALT increased | 6 (27.3) | 0 | 0 | 0 | 3 (18.8) | 4 (16.0) | 6 (24.0) | 3 (6.8) | 6 (24.0) |
| AST increased | 6 (27.3) | 13 (23.2) | 0 | 1 (3.8) | 2 (12.5) | 3 (12.0) | 6 (24.0) | 3 (6.8) | 6 (24.0) |
| Arthralgia | 4 (18.2) | 0 | 0 | 0 | 1 (6.3) | 3 (12.0) | 7 (28.0) | 6 (13.6) | 2 (8.0) |
| Myalgia | 2 (9.1) | 5 (8.9) | 5 (25.0) | 3 (11.5) | 4 (25.0) | 4 (16.0) | 5 (20.0) | 4 (9.1) | 3 (12.0) |
| Pyrexia | 5 (22.7) | 0 | 0 | 0 | 3 (18.8) | 5 (20.0) | 2 (8.0) | 5 (11.4) | 1 (4.0) |
| Weight decreased | 5 (22.7) | 13 (23.2) | 2 (10.0) | 5 (19.2) | 1 (6.3) | 3 (12.0) | 3 (12.0) | 8 (18.2) | 0 |
| Hypokalemia | 7 (31.8) | 12 (21.4) | 3 (15.0) | 3 (11.5) | 2 (12.5) | 2 (8.0) | 3 (12.0) | 3 (6.8) | 2 (8.0) |
| Dyspnea | 5 (22.7) | 9 (16.1) | 7 (35.0) | 11 (42.3) | 0 | 4 (16.0) | 2 (8.0) | 5 (11.4) | 2 (8.0) |
| Hypertension | 3 (13.6) | 4 (7.1) | 0 | 1 (3.8) | 4 (25.0) | 1 (4.0) | 3 (12.0) | 0 | 4 (16.0) |
| GGT increased | 2 (9.1) | 0 | 0 | 0 | 0 | 2 (8.0) | 1 (4.0) | 1 (2.3) | 6 (24.0) |
| Pain in extremity | 2 (9.1) | 0 | 0 | 0 | 5 (31.3) | 0 | 2 (8.0) | 1 (2.3) | 2 (8.0) |
| Anemia | 0 | 12 (21.4) | 3 (15.0) | 4 (15.4) | 0 | 0 | 0 | 0 | 0 |
| Insomnia | 0 | 8 (14.3) | 3 (15.0) | 7 (26.9) | 0 | 2 (8.0) | 2 (8.0) | 5 (11.4) | 5 (20.0) |
| **Grade ≥3 TEAEs** |  |  |  |  |  |  |  |  |  |
| ≥1 Grade ≥3 TEAE | 16 (72.7) | 40 (71.4) | 14 (70.0) | 14 (53.8) | 6 (37.5) | 19 (76.0) | 17 (68.0) | 22 (50.0) | 15 (60.0) |
| Neutropenia | 2 (9.1) | 7 (12.5) | 1 (5.0) | 0 | 1 (6.3) | 5 (20.0) | 6 (24.0) | 1 (2.3) | 5 (20.0) |
| Constipation | 4 (18.2) | 4 (7.1) | 1 (5.0) | 1 (3.8) | 3 (18.8) | 2 (8.0) | 0 | 2 (4.5) | 1 (4.0) |
| Hyponatremia | 1 (4.5) | 6 (10.7) | 1 (5.0) | 4 (15.4) | 1 (6.3) | 2 (8.0) | 1 (4.0) | 2 (4.5) | 1 (4.0) |
| Malignant neoplasm progression | 1 (4.5) | 3 (5.4) | 0 | 1 (3.8) | 0 | 4 (16.0) | 0 | 2 (4.5) | 0 |
| Pneumonia | 4 (18.2) | 2 (3.6) | 0 | 2 (7.7) | 0 | 0 | 0 | 2 (4.5) | 1 (4.0) |
| Hypophosphatemia | 2 (9.1) | 3 (5.4) | 1 (5.0) | 0 | 0 | 0 | 1 (4.0) | 3 (6.8) | 0 |
| Fatigue | 1 (4.5) | 4 (7.1) | 1 (5.0) | 1 (3.8) | 0 | 2 (8.0) | 0 | 2 (4.5) | 0 |
| Lipase increased | 3 (13.6) | 0 | 2 (10.0) | 0 | 0 | 0 | 0 | 0 | 2 (8.0) |
| Pulmonary embolism | 3 (13.6) | 4 (7.1) | 1 (5.0) | 1 (3.8) | 0 | 0 | 0 | 1 (2.3) | 1 (4.0) |
| Anemia | 0 | 5 (8.9) | 1 (5.0) | 0 | 0 | 0 | 2 (8.0) | 1 (2.3) | 1 (4.0) |
| AST increased | 0 | 2 (3.6) | 0 | 0 | 0 | 1 (4.0) | 0 | 1 (2.3) | 2 (8.0) |
| Febrile neutropenia | 0 | 2 (3.6) | 0 | 0 | 0 | 0 | 2 (8.0) | 1 (2.3) | 1 (4.0) |
| GGT increased | 0 | 3 (5.4) | 0 | 2 (7.7) | 0 | 2 (8.0) | 0 | 0 | 2 (8.0) |
| Hypertension | 0 | 2 (3.6) | 0 | 0 | 0 | 0 | 2 (8.0) | 0 | 2 (8.0) |
| Urinary tract infection | 1 (4.5) | 0 | 0 | 0 | 0 | 1 (4.0) | 0 | 2 (4.5) | 0 |
| Diarrhea | 0 | 2 (3.6) | 1 (5.0) | 0 | 0 | 2 (8.0) | 0 | 0 | 1 (4.0) |
| General physical health deterioration | 0 | 0 | 0 | 0 | 0 | 0 | 0 | 0 | 3 (12.0) |
| Hypertriglyceridemia | 0 | 1 (1.8) | 0 | 0 | 1 (6.3) | 1 (4.0) | 1 (4.0) | 0 | 0 |
| Hypokalemia | 0 | 0 | 0 | 1 (3.8) | 0 | 1 (4.0) | 0 | 1 (2.3) | 1 (4.0) |
| Leukopenia | 0 | 1 (1.8) | 0 | 0 | 0 | 0 | 1 (4.0) | 0 | 2 (8.0) |
| Lymphocyte count decreased | 0 | 1 (1.8) | 0 | 0 | 0 | 0 | 0 | 2 (4.5) | 1 (4.0) |
| Peripheral sensorimotor neuropathy | 0 | 1 (1.8) | 0 | 0 | 0 | 1 (4.0) | 1 (4.0) | 1 (2.3) | 0 |
| Small intestinal obstruction | 0 | 0 | 0 | 1 (3.8) | 0 | 0 | 0 | 1 (2.3) | 2 (8.0) |
| Abdominal pain | 0 | 2 (3.6) | 0 | 0 | 1 (6.3) | 1 (4.0) | 0 | 0 | 0 |
| ALT increased | 0 | 2 (3.6) | 0 | 0 | 0 | 1 (4.0) | 1 (4.0) | 0 | 0 |
| Amylase increased | 0 | 0 | 0 | 0 | 0 | 1 (4.0) | 0 | 0 | 1 (4.0) |
| Biliary obstruction | 0 | 0 | 0 | 0 | 0 | 0 | 0 | 1 (2.3) | 1 (4.0) |
| Hyperglycemia | 0 | 0 | 0 | 1 (3.8) | 0 | 2 (8.0) | 0 | 0 | 0 |
| Inappropriate antidiuretic hormone secretion | 0 | 0 | 0 | 0 | 0 | 1 (4.0) | 1 (4.0) | 0 | 0 |
| Neuralgia | 0 | 0 | 0 | 0 | 0 | 0 | 1 (4.0) | 1 (2.3) | 0 |
| Neutrophil count decreased | 0 | 2 (3.6) | 0 | 1 (3.8) | 0 | 0 | 0 | 0 | 0 |
| Peripheral sensory neuropathy | 1 (4.5) | 0 | 0 | 0 | 0 | 0 | 0 | 1 (2.3) | 0 |
| Pneumonia aspiration | 0 | 1 (1.8) | 0 | 0 | 0 | 0 | 2 (8.0) | 0 | 0 |
| Septic shock | 0 | 1 (1.8) | 0 | 1 (3.8) | 0 | 0 | 0 | 0 | 2 (8.0) |
| Vomiting | 1 (4.5) | 3 (5.4) | 0 | 0 | 0 | 0 | 0 | 0 | 1 (4.0) |
| Weight decreased | 1 (4.5) | 0 | 0 | 0 | 1 (6.3) | 0 | 0 | 0 | 0 |
| Acute kidney injury | 0 | 0 | 0 | 0 | 0 | 0 | 0 | 0 | 1 (4.0) |
| Acute motor-sensory axonal neuropathy | 0 | 0 | 0 | 0 | 0 | 0 | 0 | 0 | 1 (4.0) |
| Acute respiratory failure | 0 | 1 (1.8) | 0 | 0 | 0 | 0 | 0 | 0 | 0 |
| Adductor vocal cord weakness | 0 | 0 | 0 | 0 | 0 | 0 | 0 | 0 | 1 (4.0) |
| Adrenal insufficiency | 0 | 0 | 0 | 0 | 0 | 0 | 0 | 1 (2.3) | 0 |
| Arthralgia | 0 | 1 (1.8) | 1 (5.0) | 0 | 0 | 0 | 0 | 0 | 0 |
| Atrial fibrillation | 0 | 1 (1.8) | 0 | 0 | 0 | 0 | 0 | 0 | 0 |
| Back pain | 0 | 1 (1.8) | 0 | 0 | 0 | 0 | 0 | 0 | 0 |
| Bacteremia | 0 | 0 | 0 | 0 | 0 | 0 | 0 | 1 (2.3) | 0 |
| Blood alkaline phosphatase increased | 0 | 0 | 0 | 1 (3.8) | 0 | 1 (4.0) | 0 | 0 | 0 |
| Blood creatine phosphokinase increased | 0 | 1 (1.8) | 0 | 0 | 0 | 1 (4.0) | 0 | 0 | 0 |
| Bone pain | 0 | 1 (1.8) | 0 | 0 | 0 | 0 | 0 | 0 | 0 |
| Bronchitis | 0 | 0 | 0 | 0 | 0 | 0 | 1 (4.0) | 0 | 0 |
| Cancer pain | 0 | 0 | 1 (5.0) | 0 | 0 | 0 | 0 | 0 | 0 |
| Cellulitis | 0 | 0 | 0 | 0 | 0 | 1 (4.0) | 0 | 0 | 0 |
| Cerebrovascular accident | 0 | 1 (1.8) | 0 | 0 | 0 | 0 | 0 | 0 | 0 |
| Chest pain | 0 | 1 (1.8) | 0 | 1 (3.8) | 0 | 0 | 0 | 0 | 0 |
| Cholangitis infective | 0 | 0 | 0 | 0 | 0 | 1 (4.0) | 0 | 0 | 0 |
| Colitis | 0 | 3 (5.4) | 1 (5.0) | 0 | 0 | 0 | 0 | 0 | 0 |
| Decreased appetite | 0 | 2 (3.6) | 0 | 0 | 0 | 0 | 0 | 0 | 0 |
| Deep vein thrombosis | 1 (4.5) | 0 | 0 | 0 | 0 | 0 | 0 | 0 | 0 |
| Dehydration | 0 | 1 (1.8) | 1 (5.0) | 0 | 0 | 0 | 0 | 0 | 0 |
| Depressed level of consciousness | 0 | 0 | 0 | 0 | 0 | 0 | 0 | 1 (2.3) | 0 |
| Depression | 0 | 1 (1.8) | 0 | 0 | 0 | 0 | 0 | 0 | 0 |
| Device occlusion | 0 | 0 | 0 | 0 | 0 | 0 | 0 | 0 | 1 (4.0) |
| Dysphagia | 0 | 0 | 0 | 0 | 0 | 0 | 0 | 1 (2.3) | 0 |
| Dyspnea | 0 | 4 (7.1) | 1 (5.0) | 2 (7.7) | 0 | 0 | 0 | 1 (2.3) | 0 |
| Embolism | 0 | 0 | 0 | 0 | 0 | 0 | 0 | 1 (2.3) | 0 |
| Encephalopathy | 0 | 0 | 0 | 0 | 0 | 0 | 1 (4.0) | 0 | 0 |
| Fall | 0 | 0 | 0 | 0 | 0 | 0 | 0 | 0 | 1 (4.0) |
| Femoral neck fracture | 0 | 1 (1.8) | 0 | 0 | 0 | 0 | 0 | 0 | 0 |
| Hemoptysis | 1 (4.5) | 0 | 0 | 1 (3.8) | 0 | 0 | 0 | 0 | 0 |
| Hemorrhoids | 0 | 0 | 0 | 0 | 0 | 0 | 1 (4.0) | 0 | 0 |
| Hip fracture | 0 | 1 (1.8) | 0 | 0 | 0 | 0 | 0 | 0 | 0 |
| Hyperbilirubinemia | 0 | 1 (1.8) | 0 | 0 | 0 | 0 | 0 | 0 | 0 |
| Hypocalcemia | 0 | 1 (1.8) | 0 | 0 | 0 | 0 | 0 | 0 | 0 |
| Hypoglycemia | 0 | 0 | 0 | 0 | 0 | 1 (4.0) | 0 | 0 | 0 |
| Hypomagnesemia | 0 | 1 (1.8) | 0 | 0 | 0 | 0 | 0 | 0 | 0 |
| Hypotension | 0 | 4 (7.1) | 0 | 0 | 0 | 0 | 1 (4.0) | 0 | 0 |
| Ileus | 1 (4.5) | 1 (1.8) | 0 | 0 | 0 | 0 | 0 | 0 | 0 |
| Infection | 0 | 0 | 0 | 0 | 0 | 1 (4.0) | 0 | 0 | 0 |
| Intestinal obstruction | 0 | 0 | 0 | 0 | 0 | 0 | 0 | 1 (2.3) | 0 |
| Lower respiratory tract infection | 0 | 2 (3.6) | 0 | 0 | 0 | 0 | 0 | 0 | 0 |
| Lymphocyte count decreased | 0 | 1 (1.8) | 0 | 0 | 0 | 0 | 0 | 2 (4.5) | 1 (4.0) |
| Lymphopenia | 0 | 0 | 0 | 0 | 0 | 0 | 0 | 0 | 1 (4.0) |
| Metastases to central nervous system | 0 | 0 | 0 | 0 | 0 | 1 (4.0) | 0 | 0 | 0 |
| Monoplegia | 0 | 0 | 0 | 0 | 0 | 0 | 0 | 0 | 1 (4.0) |
| Multiple organ dysfunction syndrome | 0 | 0 | 0 | 0 | 0 | 0 | 1 (4.0) | 0 | 0 |
| Myalgia | 0 | 1 (1.8) | 1 (5.0) | 0 | 1 (6.3) | 0 | 0 | 0 | 0 |
| Nausea | 0 | 5 (8.9) | 1 (5.0) | 0 | 0 | 0 | 0 | 0 | 1 (4.0) |
| Neoplasm progression | 0 | 0 | 0 | 0 | 0 | 0 | 0 | 1 (2.3) | 0 |
| Pericardial effusion malignant | 1 (4.5) | 0 | 0 | 0 | 0 | 0 | 0 | 0 | 0 |
| Peripheral motor neuropathy | 1 (4.5) | 0 | 0 | 0 | 0 | 0 | 0 | 0 | 0 |
| Physical deconditioning | 0 | 0 | 0 | 0 | 0 | 1 (4.0) | 0 | 0 | 0 |
| Pleural effusion | 0 | 1 (1.8) | 1 (5.0) | 0 | 0 | 0 | 0 | 0 | 0 |
| Pneumocystis jirovecii infection | 0 | 0 | 0 | 0 | 0 | 0 | 0 | 1 (2.3) | 0 |
| Pneumonitis | 0 | 2 (3.6) | 0 | 0 | 0 | 0 | 0 | 0 | 0 |
| Pneumothorax | 0 | 0 | 0 | 0 | 0 | 0 | 1 (4.0) | 0 | 0 |
| Sepsis | 0 | 2 (3.6) | 0 | 0 | 0 | 0 | 0 | 0 | 0 |
| Staphylococcal bacteremia | 0 | 0 | 0 | 0 | 0 | 1 (4.0) | 0 | 0 | 0 |
| Supraventricular tachycardia | 0 | 1 (1.8) | 0 | 0 | 0 | 0 | 0 | 1 (2.3) | 0 |
| Tracheal obstruction extrinsic | 0 | 0 | 0 | 0 | 0 | 1 (4.0) | 0 | 0 | 0 |
| White blood cell count decreased | 0 | 1 (1.8) | 0 | 1 (3.8) | 0 | 0 | 0 | 0 | 0 |
| Urinary tract infection | 1 (4.5) | 0 | 0 | 0 | 0 | 1 (4.0) | 0 | 2 (4.5) | 0 |

Abbreviations: Q3W, once every 3 weeks; 3Q4W, 3 weekly doses every 4 weeks; ALT, alanine aminotransferase; AST, aspartate aminotransferase; GGT, gamma glutamyl transferase; NSCLC, non-small cell lung cancer; TEAE, treatment-emergent adverse event.
