# Supplementary material for: Phase I/II Study of AXL-Specific Antibody–Drug Conjugate Enapotamab Vedotin in Patients with Advanced Solid Tumors
Source: Cancer Res Commun. 2025 Nov 26;5(11):2066–78. doi: 10.1158/2767-9764.CRC-25-0359 (PMC12648153; doi:10.1158/2767-9764.CRC-25-0359)
Supplement: Table S6 — Coefficient p-values for logistic regression models of investigator confirmed ORR and DCR built from radiomic feature-derived signatur [file crc-25-0359_table_s6_suppst6.docx]

**Supplementary Table S6.** Coefficient p-values for logistic regression models of investigator confirmed ORR and DCR built from radiomic feature-derived signatures.

| **Radiomics Signature** | **ORR** | **DCR** |
| --- | --- | --- |
| Baseline (n=64) | 0.34 | 0.16 |
| Cycle 2 (n=50) | 0.33 | 0.033* |
| Longitudinal (n=48) | 0.32 | 0.03* |

*Denotes statistical significance.

Abbreviations: DCR, disease control rate; ORR, overall response rate.
