# Supplementary material for: Phase I/II Study of AXL-Specific Antibody–Drug Conjugate Enapotamab Vedotin in Patients with Advanced Solid Tumors
Source: Cancer Res Commun. 2025 Nov 26;5(11):2066–78. doi: 10.1158/2767-9764.CRC-25-0359 (PMC12648153; doi:10.1158/2767-9764.CRC-25-0359)
Supplement: Table S7 — Loading values of radiomic features for principal component 1 comprising radiomics signatures at baseline, cycle 2, and longitudinal; the top 5 contributors to each radiomics signature highlighted with colored arrows [file crc-25-0359_table_s7_suppst7.docx]

**Supplementary Table S7.** Loading values of radiomic features for principal component 1 comprising radiomics signatures at baseline, cycle 2, and longitudinal; the top 5 contributors to each radiomics signature highlighted with colored arrows.

| **Baseline** | **Contribution** | **Cycle 2** | **Contribution** | **Longitudinal** | **Contribution** |
| --- | --- | --- | --- | --- | --- |
| GLSZM Small Zone High Gray Level Emphasis | −0.328 **←** | Histogram Kurtosis | −0.2588 | GLSZM Low Grey Level Emphasis | −0.4639 **←** |
| Morphologic Roundness | −0.3085 **←** | GLSZM Small Zone High Gray Level Emphasis | −0.2392 | GLSZM Small Zone High Gray Level Emphasis | −0.0647 |
| Intensity Minimum | −0.2454 | GLCM Entropy | −0.2204 | Morphologic Roundness | −0.0267 |
| GLSZM Zone Size Entropy | −0.2042 | GLSZM Small Zone High Grey Level Emphasis | −0.2152 **←** | Intensity Mode | −0.024 |
| GLSZM Zone Size None Uniformity Normalized | −0.0731 | Intensity Standard Deviation | −0.158 | Intensity Skewness | 0.02 |
| Morphology Eccentricity | −0.0379 | Morphologic Roundness | −0.1577 | Morphologic Eccentricity | −0.0118 |
| Intensity Mean Absolute Deviation | −0.0182 | Intensity Quartile Coefficient of Variation | −0.0491 **←** | Morphologic Flatness | 0.1078 |
| Histogram Mean Absolute Deviation | −0.0117 **←** | Morphologic Eccentricity | −0.0379 | GLSZM Zone Size Entropy | 0.1733 |
| Intensity Skewness | 0.0286 | Intensity Mode | 0.0108 | Intensity Minimum | 0.2703 |
| Intensity Coefficient of Variation | 0.1033 | Intensity Range | 0.1181 | Intensity 90th Percentile | 0.3314 **←** |
| Morphologic Flatness | 0.1522 | Intensity Minimum | 0.1247 | Intensity Quartile Coefficient of Variation | 0.3655 **←** |
| Intensity Mode | 0.1606 | Histogram Mean Absolute Deviation | 0.1825 **←** | Histogram Mean Absolute Deviation | 0.4469 **←** |
| GLSZM Low Gray Level Emphasis | 0.1906 **←** | Histogram Median Absolute Deviation | 0.1885 **←** | Histogram Mean Absolute Deviation | 0.47 **←** |
| Intensity Quartile Coefficient of Variation | 0.1955 **←** | Intensity 90th Percentile | 0.2512 **←** | **←** Top 5 contributors at baseline  **←** Top 5 contributors longitudinal  **←** Top 5 contributors at Cycle 2 | |
| Histogram Median Absolute Variation | 0.2799 **←** | GLSZM Zone Size Entropy | 0.2718 |  |  |
| Intensity 90th Percentile | 0.3021 **←** | Histogram Skewness | 0.301 **←** |  |  |
| Morphologic Equivalent Spherical Radius | 0.3297 **←** | Histogram Uniformity | 0.3089 **←** |  |  |
| Intensity Mean | 0.3626 **←** | Morphologic Flatness | 0.3149 **←** |  |  |
| Histogram 95th Percentile | 0.3785 **←** | Intensity Skewness | 0.3177 **←** |  |  |
|  | | Morphologic Ellipse Diameter Axis 0 | 0.3251 **←** |  |  |

Abbreviations: GLCM, gray level co-occurrence matrix; GLSZM, gray level size zone matrix.
