# Supplementary figures and images for: Phase I/II Study of AXL-Specific Antibody–Drug Conjugate Enapotamab Vedotin in Patients with Advanced Solid Tumors
Source: Cancer Res Commun. 2025 Nov 26;5(11):2066–78. doi: 10.1158/2767-9764.CRC-25-0359 (PMC12648153; doi:10.1158/2767-9764.CRC-25-0359)

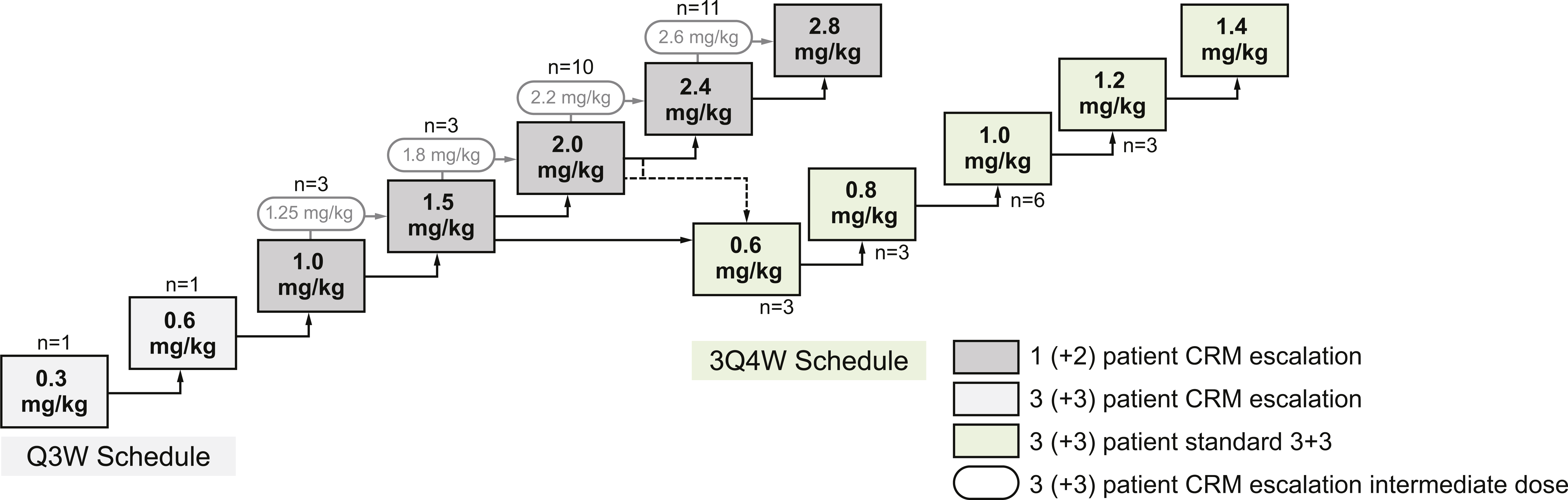

Supplement: Figure S1 — Design of dose-escalation phase. CRM, continuous reassessment model; Q3W, once every 3 weeks; 3Q4W, 3 weekly doses every 4 weeks [file crc-25-0359_figure_s1_suppsf1.png]

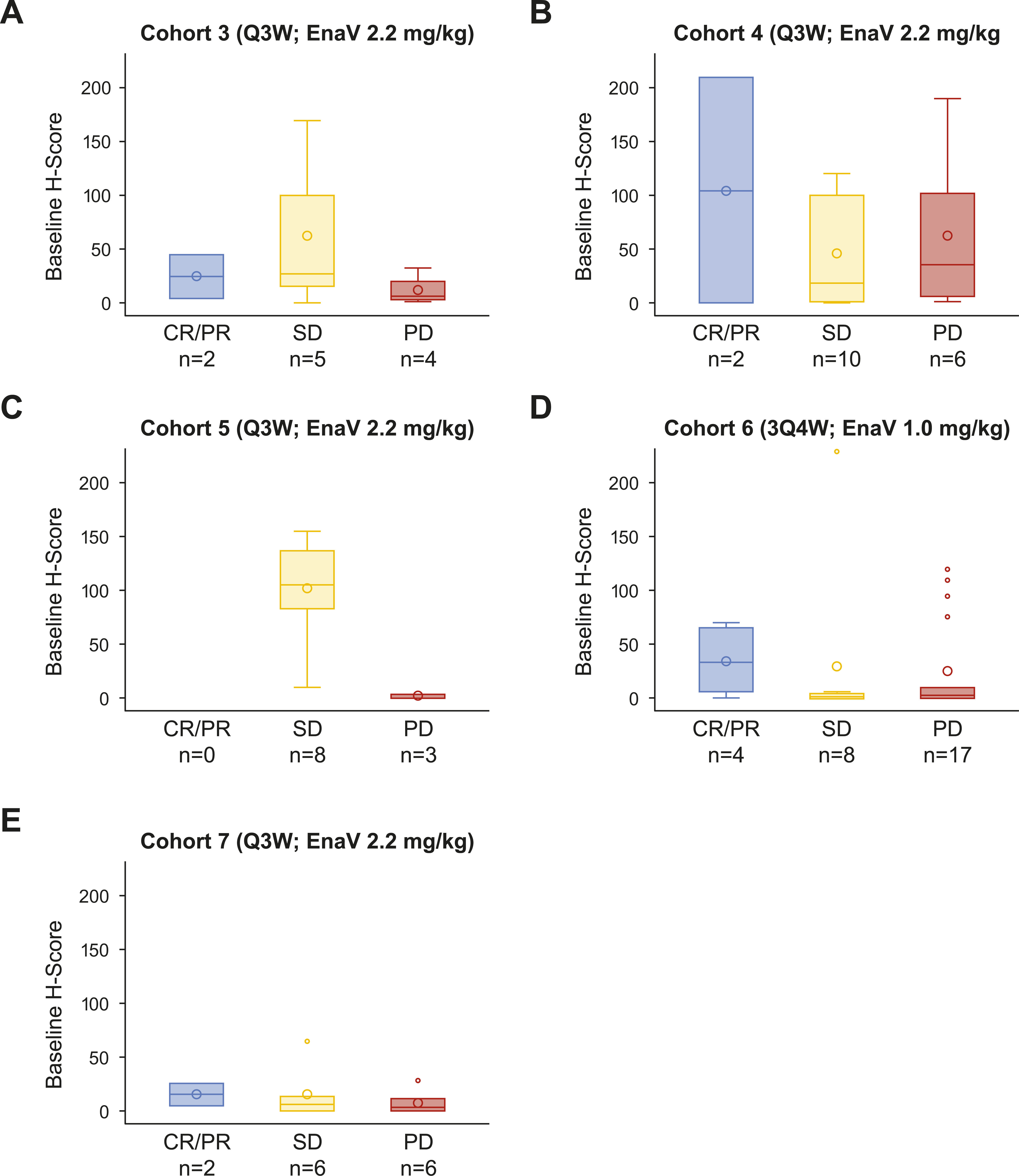

Supplement: Figure S2 — Baseline tumor H-score levels for AXL positivity by best confirmed overall response per investigator assessment in expansion cohorts. (A) Cohort 3 (Q3W): melanoma with BRAF V600 mutation; (B) cohort 4 (Q3W): melanoma with BRAF V600 wild-type; (C) cohort 5 (Q3W): sarcoma; (D) cohort 6 (3Q4W): other metastatic solid tumors; and (E) cohort 7 (Q3W): platinum-resistant ovarian cancer . CR, complete response; PD, progressive disease; PR, partial response; Q3W, once every 3 weeks; 3Q4W, 3 weekly doses every 4 weeks; SD, stable disease [file crc-25-0359_figure_s2_suppsf2.png]

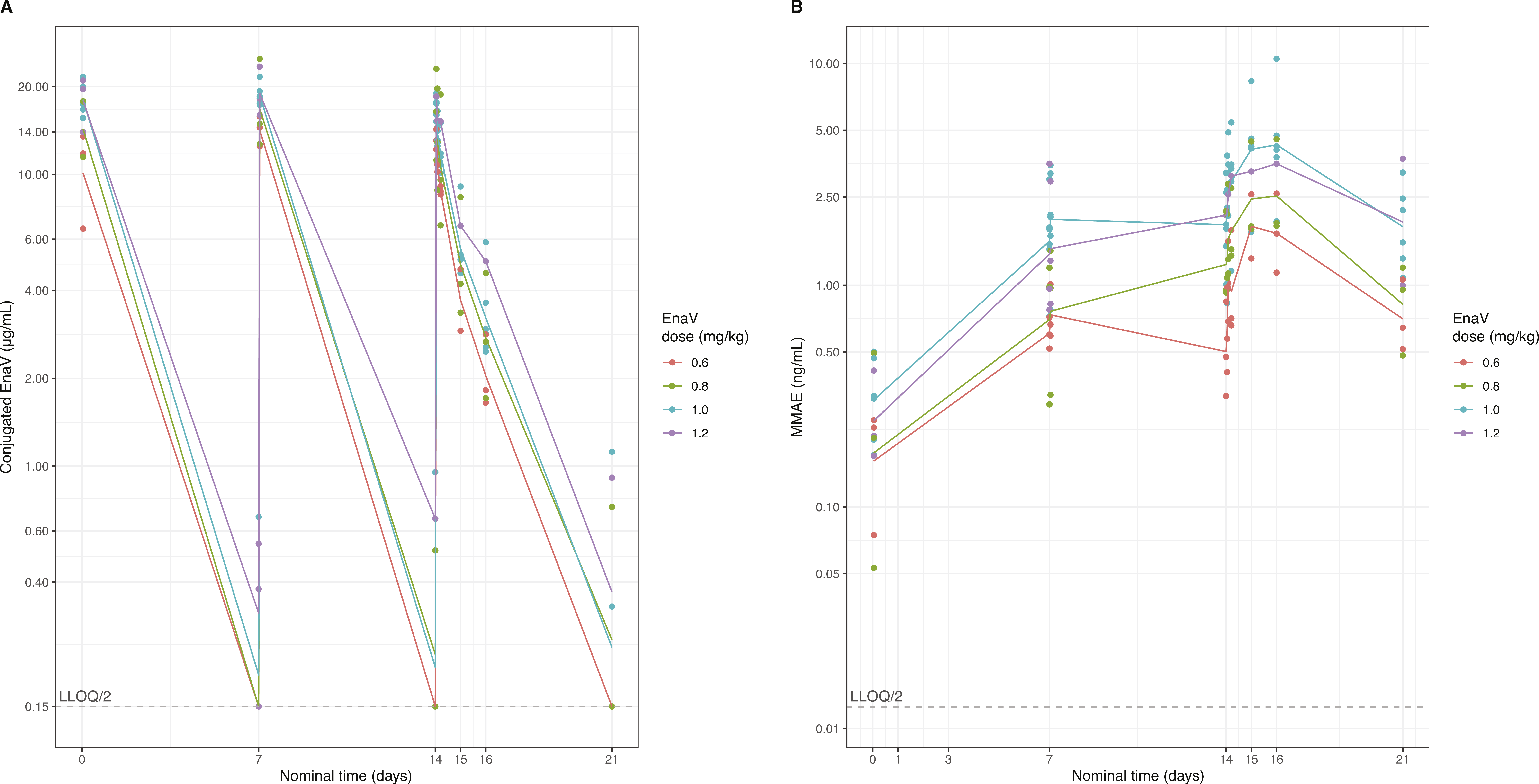

Supplement: Figure S3 — Pharmacokinetic profile for EnaV in dose-escalation phase. Plasma/serum concentrations of (A) conjugated EnaV and (B) MMAE at 3Q4W. Dots indicate observed data; lines indicate mean data. EnaV, enapotamab vedotin; LLOQ, lower limit of quantitation; MMAE, monomethyl auristatin E [file crc-25-0359_figure_s3_suppsf3.png]
